# DNA Barcoding Identifies Argentine Fishes from Marine and Brackish Waters

**DOI:** 10.1371/journal.pone.0028655

**Published:** 2011-12-09

**Authors:** Ezequiel Mabragaña, Juan Martín Díaz de Astarloa, Robert Hanner, Junbin Zhang, Mariano González Castro

**Affiliations:** 1 Laboratorio de Biotaxonomía Morfológica y Molecular de Peces, Instituto de Investigaciones Marinas y Costeras, Facultad de Ciencias Exactas y Naturales, Universidad Nacional de Mar del Plata, Mar del Plata, Argentina; 2 Consejo Nacional de Investigaciones Científicas y Técnicas, Argentina; 3 Biodiversity Institute of Ontario and Department of Integrative Biology, University of Guelph, Ontario Canada; 4 College of Fisheries and Life Science, Shanghai Ocean University, Shanghai; Ecole Normale Supérieure de Lyon, France

## Abstract

**Background:**

DNA barcoding has been advanced as a promising tool to aid species identification and discovery through the use of short, standardized gene targets. Despite extensive taxonomic studies, for a variety of reasons the identification of fishes can be problematic, even for experts. DNA barcoding is proving to be a useful tool in this context. However, its broad application is impeded by the need to construct a comprehensive reference sequence library for all fish species. Here, we make a regional contribution to this grand challenge by calibrating the species discrimination efficiency of barcoding among 125 Argentine fish species, representing nearly one third of the known fauna, and examine the utility of these data to address several key taxonomic uncertainties pertaining to species in this region.

**Methodology/Principal Findings:**

Specimens were collected and morphologically identified during crusies conducted between 2005 and 2008. The standard BARCODE fragment of COI was amplified and bi-directionally sequenced from 577 specimens (mean of 5 specimens/species), and all specimens and sequence data were archived and interrogated using analytical tools available on the Barcode of Life Data System (BOLD; www.barcodinglife.org). Nearly all species exhibited discrete clusters of closely related haplogroups which permitted the discrimination of 95% of the species (i.e. 119/125) examined while cases of shared haplotypes were detected among just three species-pairs. Notably, barcoding aided the identification of a new species of skate, *Dipturus argentinensis*, permitted the recognition of *Genypterus brasiliensis* as a valid species and questions the generic assignment of *Paralichthys isosceles*.

**Conclusions/Significance:**

This study constitutes a significant contribution to the global barcode reference sequence library for fishes and demonstrates the utility of barcoding for regional species identification. As an independent assessment of alpha taxonomy, barcodes provide robust support for most morphologically based taxon concepts and also highlight key areas of taxonomic uncertainty worthy of reappraisal.

## Introduction

Despite ongoing scientific debate concerning the role of molecular methods in taxonomy DNA barcoding has emerged as a widely accepted tool for species identification because of its enhanced focus on standardization and data validation [1]. Barcoding [2]–[4] seeks to extend species identification capabilities by using short, standardized gene regions for the efficient and cost-effective identification of eukaryotes. Advocating the use of an easily characterized 648 bP fragment from the mitochondrial 5′ region of the cytochrome *c* oxidase subunit I (COI) gene for animal identification, the primary goal of barcoding focuses on the assembly of reference sequence libraries derived from expert-identified voucher specimens in order to develop reliable molecular tools for species identification in nature [5]. Barcoding has been mischaracterized as molecular taxonomy [6], although it is not intended to replace classical taxonomy [1]. Its purpose is to facilitate species identifications by non-experts and to do so in a rapid and cost-effective manner [7]. The effectiveness of barcoding has been demonstrated in diverse taxa, including springtails [8], spiders [9], butterflies [2], [3], [10]–[12], flies [13], bivalves [14], fishes [15], birds [16], [17] and mammals [18]–[20], with barcoding systems also now being established for plants [21], macroalgae [22], and bacteria [23].

The Fish Barcode of Life campaign (FISH-BOL) [24] seeks to establish a standard reference sequence library for the molecular identification of fishes worldwide [25]. The identification process using COI sequence data for fishes is promising, as supported by recent examples of its application. DNA barcoding surveys of 207 Australian marine fish species [15] and 210 Australasian shark and ray species [26] have concluded that DNA barcoding can be used for both teleost and chondricthyan species identification. Hubert et al. [5] were able to distinguish 93% of 190 Canadian freshwaters fishes using the mitochondrial DNA COI gene. Steinke et al. [27] demonstrated that sequence variability in the barcode region permitted discrimination of 98% of 201 fish species from the Canadian Pacific. Ward and Holmes [28] analysed the DNA barcode region in 388 species of fishes, including 4 holocephali and 61 elasmobranchs showing the discrimination of 98–99% of fish species examined thus far. In addition, barcodes were subsequently used to identify marine fish larvae from Australian [29], [30] and Antarctic [31] waters.

The ichthyofauna of the Argentine continental shelf is well known due to information obtained by large foreign expeditions and local research cruises since the early twentieth Century [32]–[35], [ 36 and references therein]. However, taxonomic resolution remains elusive for some challenging groups, and new species remain to be discovered as evidenced by the ongoing description of new species [35], [37]–[42].

The aim of this study is to extend barcode coverage to Argentine marine and brackish water fishes. Because museum specimens are generally recalcitrant to DNA analyses due to of their fixation in formalin, a dedicated collecting effort of fresh material was required from Argentinean waters. We examined the patterns of barcode sequence divergence among 577 specimens identified as belonging to 125 fish species, representing nearly one third of the known fauna [33], [35], [43]. The investigation not only provides the potential use of DNA barcoding as a tool to aid traditional taxonomy in the identification of Argentinean marine fish species, but also explores the application of DNA barcodes to flag overlooked species and discusses the potential limitations inherent to the existing morphologically-based taxonomic system.

## Materials and Methods

### Ethics Statement

All tissue samples were extracted from specimens that were collected as part of other biological studies carried out with appropriate permissions from local authorities: Comisión de Investigaciones Científicas de la Provincia de Buenos Aires, Argentina. (CIC, Ref. Leg. 56/S06); Consejo Nacional de Investigaciones Científicas y Técnicas (CONICET, Fdo iBOL Argentina/2008) and Universidad Nacional de Mar del Plata, Argentina (UNMdP, EXA 407/08).

### Sampling and Taxonomic Coverage

**Figure 1:**
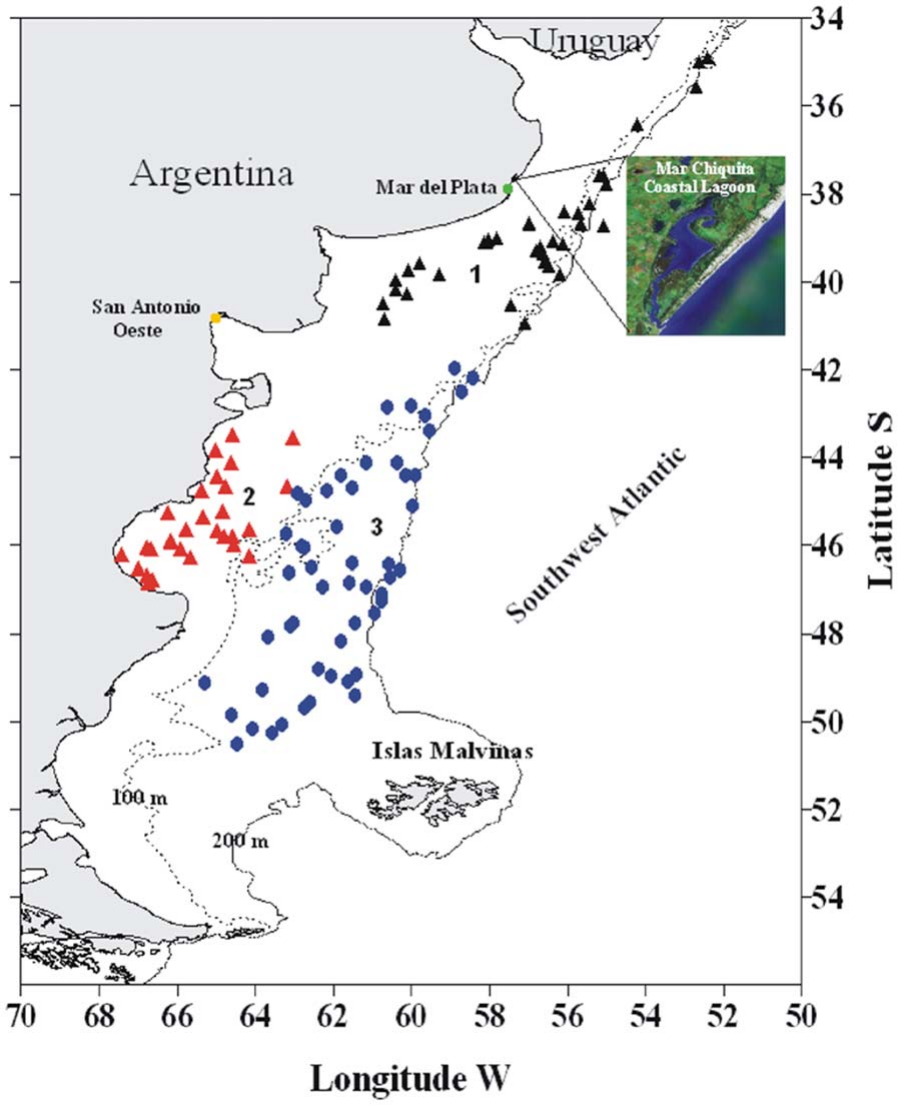
Collection sites for specimens examined in this study. Black triangles correspond to northern samples, shelf of Buenos Aires province; green circle correspond to samples collected in Mar Chiquita coastal lagoon; red triangles correspond to samples of inner shelf of Patagonian waters, and blue circles, samples of outer shelf of Patagonian waters. Numbers are provided to facilitate species collection sites of table 2.

A total of 577 specimens of fishes collected in Argentine waters, representing 125 species, 98 genera, and 63 families were analyzed. 77 of these species were collected during research cruises conducted by the National Institute for Fisheries Research and Development (INIDEP) in the Argentine Sea, whereas the other 49 species came from researches conducted by the Laboratorio de Ictiología of Universidad Nacional de Mar del Plata in Mar Chiquita coastal lagoon and off Mar del Plata coast (Figure 1, Table S1). Methods followed recommendations of FISH-BOL [24]. Vouchers were morphologically identified following the identification reliability levels 1 and 2 according to the Fish-BOL collaborator's protocol [25]; Level 1: “highly reliable identification—specimen identified by (1) an internationally recognized authority of the group, or (2) a specialist that is presently studying or has reviewed the group in the region in question” and Level 2: “identification made with high degree of confidence at all levels—specimen identified by a trained identifier who had prior knowledge of the group in the region or used available literature to identify the specimen”. Some of the specimens were deposited in the fish collection of INIDEP; the other specimens studied were kept only as e-voucher (See Table S1). All sequence assemblies, electropherogram (trace) files, primer sequences and specimen provenance data were deposited in the publicly accessible “Fishes of Argentina” Project (code FARG) on the Barcode of Life Database (BOLD, [44]). This included digital images of morphological voucher specimens, sex and ontogenetic stage (post larval, juvenile or adult), total and standard length as well as GPS coordinates for all specimen collection localities.

### DNA Analysis

Muscle tissue samples were taken from whole specimens and genomic DNA extracted according to the protocol of Ivanova et al. [45]. Amplification of the 5′ barcode region of COI was first attempted using FF2d_t1/FR1d_t1 primer combination and C_FishF1t1/C_FishR1t1 primer cocktails [46]. The primer combination C_FishF1t1 contained two primers (FishF2_t1/VF2_t1), and C_FishR1t1 also contained two primers (FishR2_t1/FR1d_t1) (Table 1). All primers were appended with M13 tails to facilitate sequencing. PCR reactions were performed in 96-well plates. The reaction master mix consisted of 825 µl water, 125 µl 10× buffer, 62.5 µl MgCl_2_ (25 mM), 6.25 µl dNTP (10 mM), 6.25 µl each primer (0.01 mM) and 6.25 µl Taq DNA polymerase (5 U/µl) was prepared for each plate, and each well contained 10.5 µl mixture and 2 µl genomic DNA. The PCR reaction profile was comprised of an initial step of 2 min at 95°C and 35 cycles of 30 sec at 94°C, 40 sec at 52°C, and 1 min at 72°C, with a final extension at 72°C for 10 min. Amplicons were visualized on 2% agarose E-Gel® 96-well system (Invitrogen). Sequencing reactions used M13 forward and reverse primers using the BigDye® Terminator v.3.1 Cycle Sequencing Kit (Applied Biosystems, Inc.), and the reaction profile was comprised of an initial step of 2 min at 96°C and 35 cycles of 30 sec at 96°C, 15 sec at 55°C, and 4 min at 60°C. Products were directly sequenced using an ABI 3730 capillary sequencer according to manufacturer's instructions. For specimens that failed to amplify using the primer combinations above, C_VF1LFt1/C_ VR1LRt1 primer combinations [46] were tried. C_VF1LFt1 consisted of four primers (VF1_t1/VF1d_t1/LepF1_t1/VFli_t1), and C_VR1LRt1 also comprised four primers (VR1_t1/VR1d_t1/LepR1_t1/VRli_t1) (Table 2). The PCR reaction profile consisted of 1 min at 95°C and 35 cycles of 30 sec at 94°C, 40 sec at 50°C, and 1 min at 72°C, with a final extension at 72°C for 10 min. All other procedures followed those above. DNA sequences were aligned with SeqScape v.2.1.1 software (Applied Biosystems, Inc.). Sequence divergences were calculated using the Kimura two parameter (K2P) distance model [47], and unrooted NJ phenograms based on K2P distances were created using BOLD. K2P model was used because data set covers a large range of taxa spanning many orders and mtDNA is subject to mutational saturation at this level. Eventhough there are several distance models that take into account this issue, K2P is one of the simplest and commonest model used for describing differentiation among species using COI. On the other hand, being K2P the standard model used in barcode studies allows a better comparison with other barcode studies. Other NJ trees analysing individual genera were constructed using MEGA 4.1 [48] and were bootstrapped 500 times to provide percentage bootstrap values for branch points. Sequence data are available on both BOLD and GenBank (Table S2).

**Table 1:** Polymorphic nucleotide sites in the COI, haplotype designation (Hd) and absolute haplotype frequency (n), for specimens of *Psammobatis normani* and *P. rudis*.

Hd	n	Position				
		133	160	457	493	501
*P. normani*	3	T	T	T	G	T
*P. rudis* A	8	.	.	.	.	.
*P. rudis* B	5	.	.	C	.	.
*P. rudis* C	3	.	.	.	A	.
*P. rudis* D	1	.	G	C	.	.
*P. rudis* E	1	A	.	.	.	.
*P. rudis* F	1	.	.	.	.	G
*P. rudis* G	1	T	T	T	G	T

**Table 2:** Primers used for amplification of fishes from Argentine.

Primer combination	Primer designation	Nucleotide
	FF2d_t1	5′TGTAAAACGACGGCCAGTTTCTCCACCAACCACAARGAYATYGG 3′
C_FishF1t1	FishF2_t1	5′TGTAAAACGACGGCCAGTCGACTAATCATAAAGATATCGGCAC 3′
	VF2_t1	5′TGTAAAACGACGGCCAGTCAACCAACCACAAAGACATTGGCAC 3′
C_FishR1t1	FishR2_t1	5′CAGGAAACAGCTATGACACTTCAGGGTGACCGAAGAATCAGAA 3′
	FR1d_t1	5′CAGGAAACAGCTATGACACCTCAGGGTGTCCGAARAAYCARAA 3′
C_VF1LFt1	VF1_t1	5′TGTAAAACGACGGCCAGTTCTCAACCAACCACAAAGACATTGG 3′
	VF1d_t1	5′TGTAAAACGACGGCCAGTTCTCAACCAACCACAARGAYATYGG3′
	LepF1_t1	5′TGTAAAACGACGGCCAGTATTCAACCAATCATAAAGATATTGG3′
	VFli_t1	5′TGTAAAACGACGGCCAGTTCTCAACCAACCAIAAIGAIATIGG3′
C_VR1LRt1	VR1_t1	5′CAGGAAACAGCTATGACTAGACTTCTGGGTGGCCRAARAAYCA3′
	VR1d_t1	5′CAGGAAACAGCTATGACTAGACTTCTGGGTGGCCAAAGAATCA 3′
	LepR1_t1	5′CAGGAAACAGCTATGACTAAACTTCTGGATGTCCAAAAAATCA3′
	VRli_t1	5′CAGGAAACAGCTATGACTAGACTTCTGGGTGICCIAAIAAICA3′

*M13 tails are underlined.* Some of the primers were already published by [46].

## Results and Discussion

### Overall results

No stop codon, insertions or deletions were found in any of the amplified sequences, showing that all of them constitute functional mitochondrial COI sequences. Of the 651 bases, 354 nucleotid sites were variable and most substitutions occurred in the third nucleotide position within codons (61.3%). All the amplified sequences were larger than 600-bp, the limit typically observed for NUMTs (nuclear DNA sequences originating from mtDNA) [49]. As reported by Ward et al [24], in FISHBOL review, NUMTs do not appear to be a concern for fish barcoding.

**Figure 2:**
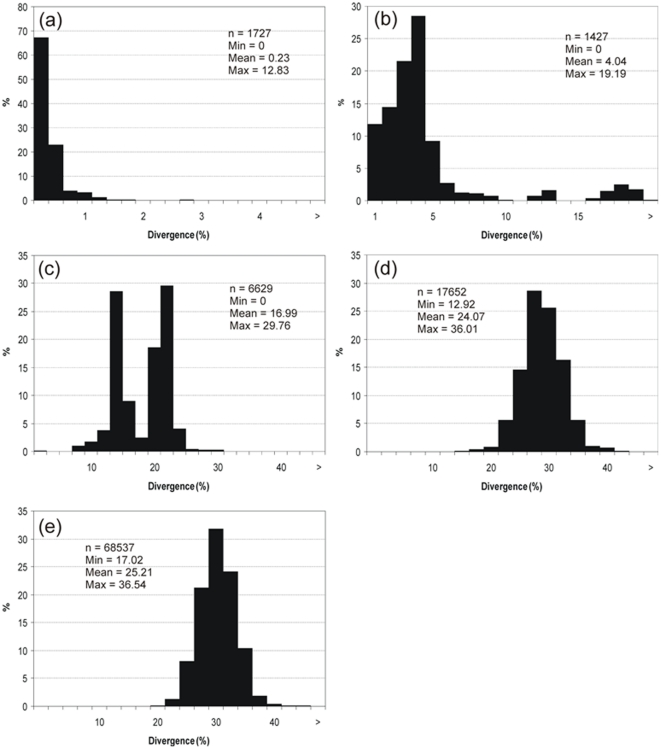
Distribution of K2P distances (%) for COI within species (a), genera (b), families (c), orders (e) and classes (f). References: n = number of comparison, Min = minimum K2P distance, Mean = mean K2P distance, Max = maximum K2P distance expressed as percentage.

A total of 577 specimens, representing 125 species, 98 genera and 63 families were barcoded (Table S1). Average nucleotide frequency were C (27.25%), T (30.74%), A (24.04%) and G (17.97%). Nearly all species exhibited unique barcode haplotypes or cohesive clusters of very closely related haplotypes, which permitted the discrimination of at least 95.2% of species (Appendix S1). The K2P genetic distances averaged just 0.23% within species, but averaged 4.04% within genera, 16.99% within families, 24.07% within orders and 25.21% within classes, increasing value with taxonomic level (Figure 2). Hence, overall, there was a 17-fold more pronounced difference among congeneric species than among conspecific individuals.

**Figure 3:**
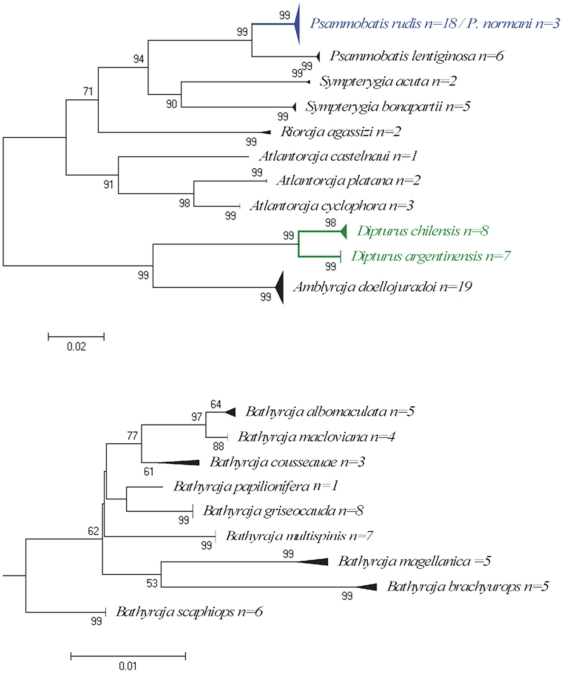
Neighbour-joining tree of COI sequences from species of the family Rajidae. Solid triangles represent clusters of multiple specimens, with the vertical dimension proportional to the number of specimens, and the horizontal depth proportional to the genetic variation within that cluster. Blue indicates clusters of specimens that include more than one species that could not be resolved. Green indicates taxa with deep intra-specific divergence that represents distinct species. Number at nodes represent bootstrap values, (only values greater than 50 are given). Due to lower variability between some species, the Genus *Bathyraja* were separated to facilitate observation.

**Figure 4:**
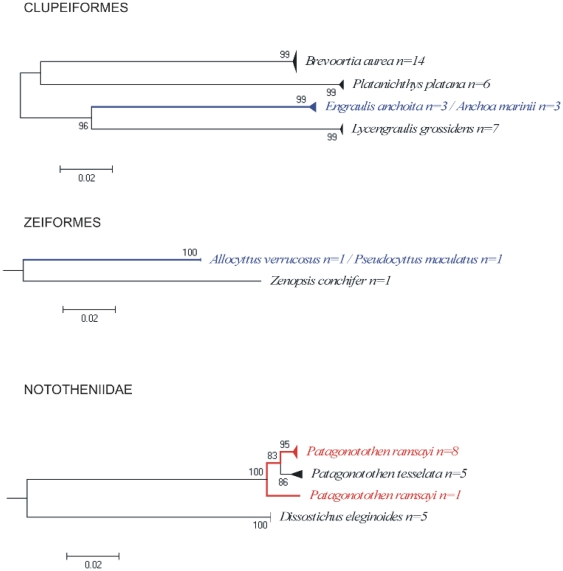
Neighbour-joining tree of COI sequences from species of the orders Clupeiformes and Zeiformes and family Nototheniidae (Perciformes). Solid triangles represent clusters of multiple specimens, with the vertical dimension proportional to the number of specimens, and the horizontal depth proportional to the genetic variation within that cluster. Blue indicates clusters of specimens that include more than one species that could not be resolved. Red indicates taxa with deep intra-specific divergence that potentially represents distinct species (see text for details). Number at nodes represent bootstrap values, (only values greater than 50 are given).

Cases of shared haplotypes were detected in six of the species analyzed, including the following pairs: the skates *Psammobatis rudis* and *P. normani*, the anchovies *Engraulis anchoita* and *Anchoa marinii*, and between the oreos *Allocyttus verrucosus* and *Pseudocyttus maculatus* (Figures 3, 4). Results observed within the genus *Psammobatis* will be discuss later. The anchovies *Engraulis anchoita* (n = 3) and *Anchoa marinii* (n = 3), could not be separated by COI even though they are two valid species of Engraulidae. These species have external morphological features that allow differentiating each other; however when specimens are not well preserved it is more difficult to identify them from images. Unfortunately these specimens were not retained and only the e-vouchers (digital images) have been recorded to reliable re-checked the identification. From images it can be seen that specimens identified as *Engraulis anchoita* clearly represent this species because of the broad silver stripe along flank, the large size of the specimens (>14 cm TL), and the large eye size in relation to snout. However those specimens identified as *Anchoa marini* are not well preserved (most lateral scales are missing, and both dorsal and anal fins can not be recognized), so it is difficult from images to identify them properly. Therefore, the lack of discrimination at molecular level between these pairs of species is likely a problem of misidentification. Regarding the zero divergence between Oreosomatid species from Argentina, this case is likely to be a misidentification. *Allocyttus verrucosus* and *Pseudocyttus maculatus* are the Oreosomatid species recorded in the southwest Atlantic [33], [50], [51]. Re-examination of e-vouchers allow identifying both specimens as *Allocyttus verrucosus*, because in both the first dorsal spine is much shorter than the second spine (the opposite occurred in *P. maculatus*). On the other hand, Ward, [52] in a general analysis of DNA barcoding divergence among fishes worldwide, showed that specimens of *Allocyttus verrucosus*, *Allocyttus niger* and *Pseudocyttus maculatus* from the Indian Ocean and Tasmanian waters were clearly identified using COI sequences and were separated from each other in the NJ tree. However, specimens of *A. verrucosus* and *P. maculatus* from Argentina clustered together and near those of *A. niger* from Indian Ocean. Therefore it is possible that both individuals from Argentina are actually *A. niger*, a species that had never been reported for the southwest Atlantic Ocean [33], [50], [51] although Yearsley et al [53] in a book of Australian seafood shows a point in the southwest Atlantic as part of the distribution of this species. Further morphological and molecular studies are needed to clarify the actual taxonomic identification of these nominal species.

The maximum intraspecific genetic variation was below 1% in 93.6% of the species analysed except for the following species: *Cynoscion guatucupa* (1.72%, n = 5), *Squalus acanthias* (1.71%, n = 11), *Helicolenus lahillei* (1.24%, n = 13), *Parona signata* (1.09%, n = 6), *Iluocoetes fimbriatus* (1.09%, n = 11) and *Prionotus nudigula* (1.09%, n = 4). In addition, deep intraspecific divergences were found between individuals of *Prepilus paru* (12.3%, n = 2), this result will be discuss later. As observed by Hebert et al. [3], species congeneric K2P distances were much higher than 2%, except between some species of the genus *Bathyraja*, but each of these species formed cohesive units and were separated from each other in the NJ tree (Figure 3). From the nine species of *Bathyraja* barcoded, only three of them presented divergent values >2%, whereas in the remain six, values were relatively lower, being the lowest within *B. albomaculata* and *B. macloviana* (D = 0.61%) (Figure 3). The relatively low divergence found among species of *Bathyraja* was also found by Spies et al. [54] studying the genetic variability in 15 species of skates from Alaska. From the 12 species of the genus *Bathyraja*, four presented distances values relatively low (D<1%), even though all are morphologically different species.

### Comments on some individual genera

#### Chondrichthyes

The use of barcodes within an integrative taxonomic framework confirmed the identification of a new species of longnose skate (*Dipturus argentinensis*) from the Argentine Sea [40]. This work included, besides typical morphological features, the COI sequence as part of the description of the new species. The seven specimens of *D. argentinensis* examined possess a unique haplotype that was substantially divergent from all other *Dipturus* species represented in Barcode of Life Data Systems (n = 15 described+5 undescribed species). Individuals of the other two species that inhabit the Argentinean waters (*D. chilensis* and *D. trachyderma*) were attempted to be sequenced, but only the former amplified, even though protocols used were the same. The failure of COI to amplify in *D. trachyderma* could be the result of mutational differences in the primer sites, indicating that *D. trachyderma* possesses a significantly divergent COI haplotype of its own [40]. Resulting NJ tree for the two species sequenced are shown in Figure 3.

**Figure 5:**
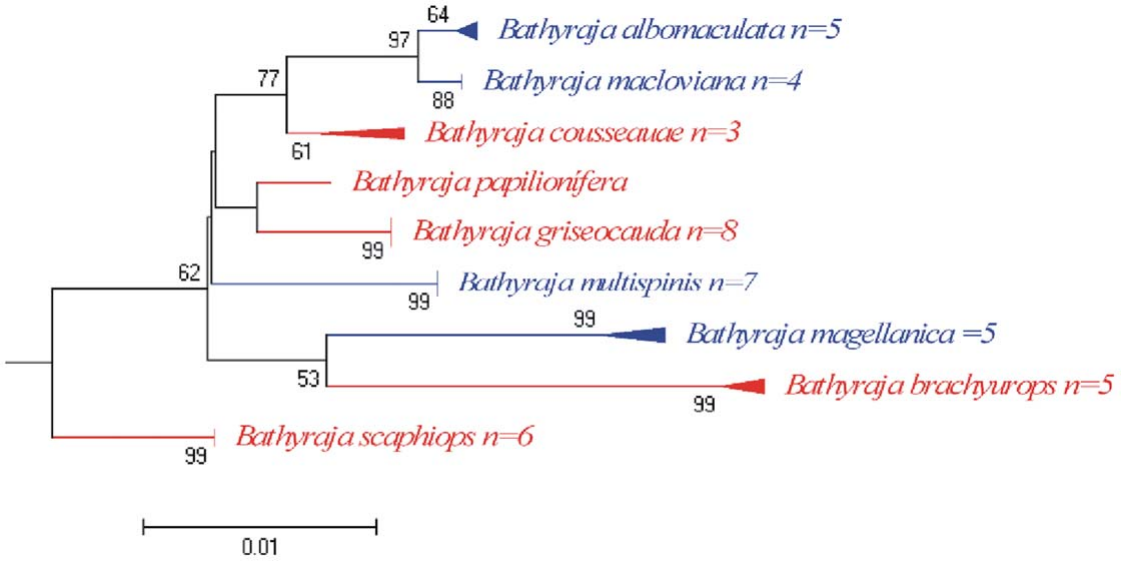
Neighbour-joining tree of COI sequences from species of *Bathyraja* from Argentina sea. Species in red indicates those assigned by Compagno [56] to the genus *Bathyraja*, where those in blue represent species assigned to the genus *Rhinoraja* (see text for details) Number at nodes represent bootstrap values, (only values greater than 50 are given).

The genus *Bathyraja* is represented in the southwestern Atlantic by 10 species [55], eight of which are endemic on the Argentinean continental shelf [34]; [38]. Compagno [56], based on osteological characteristics of rostral cartilage, assigned some species, situated in the genus *Bathyraja* by several authors [33], [55], [57], [58] to *Rhinoraja* Ishiyama. Analysis of COI sequences of “*Bathyraja*-like” species from Argentina permitted the discrimination of all them. However, species of the genus *Bathyraja* and those assigned to the genus *Rhinoraja* were clustered together, mixed and not formed two distinct groups (Figure 5). This result are not congruent to the proposal of Compagno [56], but need to be clarified trough molecular and taxonomic phylogenetic analysis that are beyond the aim of this study.

**Figure 6:**
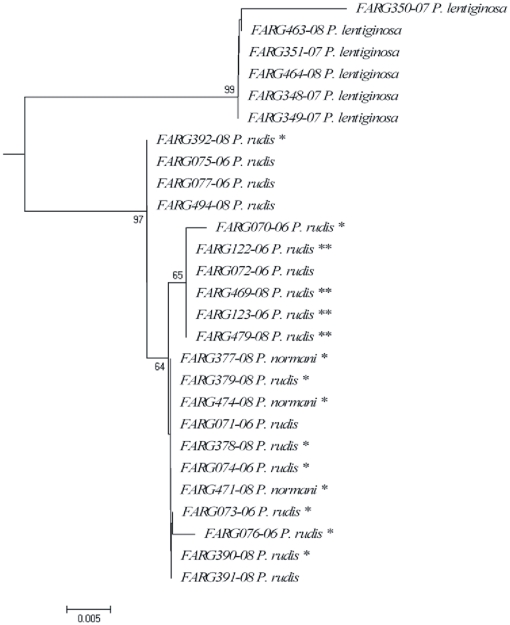
Neighbour-joining tree of *Psammobatis* COI sequences from Argentine sea. Number at nodes represent bootstrap values, (only values greater than 50 are given). Reexamination of e-voucher allow to identify clearly some specimens as *P. normani* (*) and others as *P. rudis* (**). The remaining could be either one or another.

Within the skates of the genus *Psammobatis*, specimens belonging to three different species were barcoded: *P. lentiginosa*, *P. rudis* and *P. normani*. Figure 6 showed the NJ tree for species of this genus. Only *P. lentiginosa* formed a cohesive cluster and presented a deep divergence with the other species (D = 3.62%), whereas the other two species were clustered together. All the individuals of *P. normani* (n = 3) shared the same haplotype, whereas the 18 specimens of *P. rudis* presented six different haplotypes. Six individuals possessed the same haplotype of *P. normani* and the remaining had other haplotypes with one or two different changes in nucleotides (Table 1). These two skate species are very similar in their external morphological features, especially coloration, so they are very difficult to identify. Nevertheless, both are regarded valid nominal species and could be differentiated each other by skeletal structures, such as *clasper*, *neurocranium* and *scapulocoracoid* [59], by egg cases produced by females [60] and by pattern of spinulaton [59]–[60]. Reexamination of e-vouchers made by the senior author, specialist in *Psammobatis* species, who did not participate in the research cruises in which specimens of *Psammobatis* were collected, allows to clearly identify some specimens as *P. normani* and *P. rudis* (Figure 6), but unfortunately, neither of the analyzed specimens were kept to reliable re-check the identification of those in which photographs do not show sharply distinctive features (i. e claspers, spinulation in tail etc).

The inability of barcoding to discriminate *P. rudis* and *P. normani* could be a problem of mislabeling, misidentification or could represent recent speciation. Very recently radiated species can not be separated using COI and others more rapidly evolving markers are required to identify these species [24].

Within the skates of the genus *Amblyraja*, some specimens appear as *Amblyraja* sp. because they have different color pattern from the typical *A. doellojuradoi* (Appendix S1). However, the analysis of COI revealed a random grouping of individuals and the genetic distance values are small enough to consider (conclude) that they are distinct species. Further morphological and molecular studies are needed to corroborate the statement mentioned above.

#### Actinopterygii

Figure 4 shows the neighbour-joining tree of Nototheniid species barcoded. All specimens of *Dissostichus eleginoides* and that of *Patagonotothem tesselata* formed cohesive units and were separated from each other in the NJ tree. However, within *Patagonotothen ramsayi* two clusters were observed. The first group is composed by eight specimens with 0 to 0.46% divergences, and the second group is composed by a single individual. Genetic divergence between this specimen and those of the first group ranged from 2.5 to 2.82%. The Family Nototheniidae is very diverse in Argentine waters and within the genus *Patagonotothen* 14 species have been recorded. Some of them are very difficult to recognize. The divergence between the two groups of *P. ramsayi* possibly represents a misidentification. Because we only retained the e-voucher, in which no diagnostic distinctive features are observed, we could not confirm the actual specimen identification.

**Figure 7:**
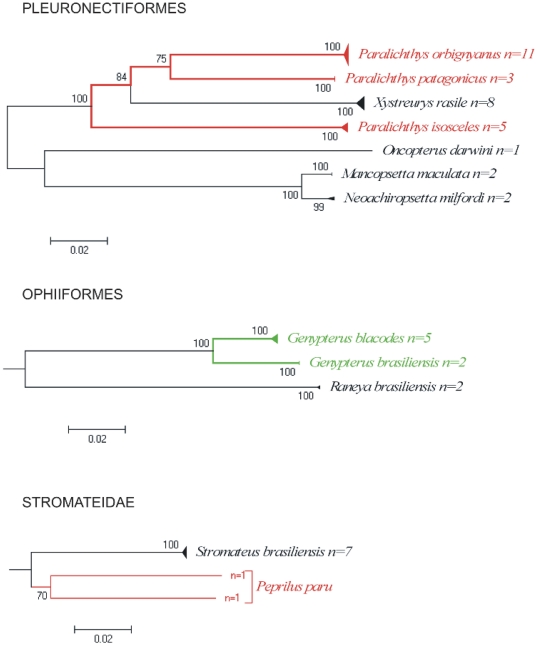
Neighbour-joining tree of COI sequences from species of the orders Pleuronectiformes and Ophidiiformes, and from the family Stromateidae (Perciformes). Solid triangles represent clusters of multiple specimens, with the vertical dimension proportional to the number of specimens, and the horizontal depth proportional to the genetic variation within that cluster. Green indicates taxa with deep intra-specific divergence that represents distinct species. Red indicates taxa with deep intra-specific divergence that potentially represents distinct species or genera (see text for details). Number at nodes represent bootstrap values, (only values greater than 50 are given).

On the other hand, the use of barcode permitted the recognition of flatfish species present in Argentine waters. The NJ tree of Pleuronectiformes revealed deep divergences between some species of the genus *Paralichthys* (Figure 7). Two of them, *P. orbignyanus* and *P. patagonicus*, are cluster together; whereas a third species (*P. isosceles*) formed a cohesive cluster located far away from the other *Paralichthys*. Moreover, genetic distance between these two groups is 16.2%, well above typical “within genera” values. Díaz de Astarloa and Munroe (in preparation) based on morphological features propose the reassignment of *P. isosceles* into a new genus. Present results on COI analysis are in accordance with them, but need to be supported by performing phylogenetic analysis that is beyond the aim of this study.

Figure 7 also shows the neighbour-joining tree obtained from specimens of cusk eels from the SW Atlantic (Ophidiiformes). Each of the two nominal species, the Brazilian cusk eel *Genypterus brasiliensis* Regan, 1903 and the Pink cusk-eel *G. blacodes* (Forster, 1801) formed cohesive units and were separated from each other in the NJ tree showing a genetic divergence of 5.8%, suggesting that both should be regarded as two different species. Díaz de Astarloa and Figueroa [61], based on morphological and osteological analysis, stated that the cusk eels *Genypterus brasiliensis* and *G. blacodes* are different species. However in the FAO species catalogue ‘Ophidiiform fishes of the world’ [62] and in the Catalogue of Fishes [63] *Genypterus brasiliensis* appears as a junior synonym of *G. blacodes*. Present results based on analysis of COI showed a deep genetic divergence between the two species. Thus, both morphological and molecular approaches strongly support that *Genypterus blacodes* and *G. brasiliensis* should be considered as valid species.

Finally, DNA barcode strongly suggest a likely second species of American harvestfish different from *Peprilus paru* (Figure 7). The kimura 2 parameter distance value was 12.83%, well above a conspecific value. In order to avoid the possibility of a mislabeling result, one individual (*Peprilus paru* FARG 563-09) was resampled and two new individuals were sampled. Results corroborate previous findings, so further morphological (e.g. meristics, traditional/landmarks-based morphometry and osteology) and molecular examination is needed to support this hypothesis.

Although some groups are highlighted for further taxonomic analysis and several species remain to be barcoded, these results support the utility of DNA barcodes for regional species identification of fishes. When comparing these results to other projects on BOLD, standardizing the application of names across collections/regions emerges as a significant challenge for FISH-BOL. However, we conclude this long-standing issue is most efficiently addressed through DNA barcoding.

## Supporting Information

Table S1 **List and number of Argentinean fishes barcoded, arranged by taxonomic category.** Clasification follows Nelson [64]. Collection sites: MdP coast, coast of Mar del Plata city; SAO coast, coast of San Antonio Oeste; MCh lagoon, Mar Chiquita coastal lagoon; 1, Off Buenos Aires province; 2, Inner shelf of Patagonian waters; 3, outer shelf of Patagonian waters. See Figure 1 for location in map. * Indicates species with voucher specimens deposited.
(PDF)

Table S2 **Details of species and specimens.** BOLD specimen numbers given, along with GenBank accession numbers, geographic locality and voucher details.
(PDF)

Appendix S1 **Neighbour-joining tree of 577 COI sequences from the 125 fish species sampled as obtained in BOLD, using K2P distances.**
(PDF)
