# Supplementary material for: DNA Barcoding Identifies Argentine Fishes from Marine and Brackish Waters
Source: PLoS One. 2011 Dec 9;6(12):e28655. doi: 10.1371/journal.pone.0028655 (PMC3235135; doi:10.1371/journal.pone.0028655)
Supplement: Table S1 — List and number of Argentinean fishes barcoded, arranged by taxonomic category. Clasification follows Nelson [64]. Collection sites: MdP coast, coast of Mar del Plata city; SAO coast, coast of San Antonio Oeste; MCh lagoon, Mar Chiquita coastal lagoon; 1, Off Buenos Aires province; 2, Inner shelf of Patagonian waters; 3, outer shelf of Patagonian waters. See Figure 1 for location in map. * Indicates species with voucher specimens deposited. (PDF) [file pone.0028655.s001.pdf]

Class<br>Order	Family	Species	Collection site					
			MdP coast	SAO Coast	MCh lagoon	1	2	3
**Actinopterygii**								
Anguilliformes	Congridae	*Bassanago albescens*						7
		Conger orbignyanus	5					
Atheriniformes	Atherinopsidae	*Odontesthes argentinensis*			8			
		*Odontesthes platensis**			1			
Batrachoidiformes	Batrachoididae	*Porichthys porosissimus*	1					
		Triathalassothia argentina	3					
Characiformes	Characidae	*Oligosarcus jenynsii*			5			
Clupeiformes	Clupeidae	*Brevoortia aurea*			15			
		Platanichthys platana			6			
	Engraulidae	*Anchoa marinii*	3					
		Engraulis anchoita					3	
		Lycengraulis grossidens			7			
	Anablepidae	*Jenynsia multidentata*			10			
	Poeciliidae	*Cnesterodon decemmaculatus*			1			
Gadiformes	Gadidae	*Micromesistius australis*						7
	Macrouridae	*Coelorinchus fasciatus*						2
		Coelorinchus marinii				6		
		Macrourus holotrachys						6
	Merlucciidae	*Macruronus magellanicus*						5
		Merluccius australis						7
		Merluccius hubbsi					5	5
	Moridae	*Notophycis marginata*						4
		Salilota australis						2

	Phycidae	*Urophycis brasiliensis*	1					
		Urophycis cirrata				1		
Myctophiformes	Myctophidae	*Gymnoscopelus nicholsi*						8
		Gymnoscopelus piabilis						1
Ophidiiformes	Ophidiidae	*Genypterus blacodes*					5	
		Genypterus brasiliensis					2	
		Raneya brasiliensis					2	
Perciformes	Blenniidae	*Hypleurochilus fissicornis*	1					
	Bovichtidae	*Bovichtus chilensis*	4					
		Cottoperca gobio						4
	Bramidae	*Brama brama*				5		
	Carangidae	*Parona signata*					6	
		*Selene setapinnis**	1					
		*Selene vomer**	1					
		Trachurus lathami	1			1		
	Centrolophidae	*Icichthys australis*						1
		Seriolella caerulea						2
		Seriolella porosa					4	
	Cheilodactylidae	*Nemadactylus bergi*					7	
	Eleginopidae	*Eleginops maclovinus*		3			4	
	Gobiidae	*Gobiosoma parri*			5			
	Malacanthidae	*Lopholatilus villarii*				1		
	Mugilidae	*Mugil platanus*			6			
	Mullidae	*Mullus argentinae*	5					
	Nototheniidae	*Dissostichus eleginoides*						6
		Patagonotothen ramsayi				5		4
	Notothenidae	*Patagonotothen tessellata*				1		4
	Percophidae	*Percophis brasiliensis*	1			4	3	

	Pinguipedidae	*Pinguipes brasilianus*			1	1	
		Pseudopercis semifasciata			1	5	
	Polyprionidae	*Polyprion americanus*	3				
	Pomatomidae	*Pomatomus saltatrix*		5			
	Sciaenidae	*Cynoscion guatucupa*		5			
		Menticirrhus americanus	5				
		Micropogonias furnieri		8			
		Paralonchurus brasiliensis	3				
		Pogonias cromis		11			
	Scombridae	*Allothunnus fallai*				1	
		Scomber japonicus			5		
	Serranidae	*Acanthistius brasilianus*	1			7	
		Serranus auriga	2				
	Sparidae	*Pagrus pagrus*			5		
	Sphyraenidae	*Sphyraena guachancho*	1				
	Stromateidae	*Peprilus paru*	2				
		Stromateus brasiliensis				7	
	Zoarcidae	*Austrolycus laticinctus*					4
		Iluocoetes fimbriatus			3	4	4
Pleuronectiformes	Achiropsettidae	*Mancopsetta maculata*					2
		Neoachiropsetta milfordi					2
	Paralichthyidae	*Paralichthys isosceles*			2	3	
		Paralichthys orbignyanus		11			
		Paralichthys patagonicus			3		
		Xystreurys rasile			2	6	
	Pleuronectidae	*Oncopterus darwini**		1			
Scorpaeniformes	Congiopodidae	*Congiopodus peruvianus*				7	
	Dactylopteridae	*Dactylopterus volitans*	1				

		Psychrolutidae	*Psychrolutes marmoratus*						1
		Sebastidae	*Helicolenus lahillei*	13					
			Sebastes oculatus					5	
		Triglidae	*Prionotus nudigula*				4		
			Prionotus punctatus	2					
	Siluriformes	Callichthyidae	*Corydoras paleatus*			2			
		Heptapteridae	*Rhamdia sapo*			1			
	Syngnathiformes	Centriscidae	*Notopogon fernandezianus*				2		
		Syngnathidae	*Hippocampus patagonicus*		6				
	Tetraodontiformes	Tetraodontidae	*Sphoeroides pachygaster*				3		
	Zeiformes	Oreosomatidae	*Allocyttus verrucosus*	1					
			Pseudocyttus maculatus					1	
		Zeidae	*Zenopsis conchifera*	1					
**Myxinii**		Myxinidae	*Myxine australis*						1
			Notomyxine tridentiger						1
**Chondrichthyes**									
	Chimaeriformes	Callorhinichidae	*Callorhinchus callorynchus*			1		8	
	Carcharhiniformes	Scyliorhinidae	*Schroederichthys bivius*				2	6	
		Triakidae	*Galeorhinus galeus*					1	
			Mustelus schmitti				1	2	
	Hexanchiformes	Hexanchidae	*Notorynchus cepedianus*	1					
	Squaliformes	Squalidae	*Squalus acanthias*				3	4	4
			Squalus mitsukurii				2		
	Squatiniformes	Squatinidae	*Squatina guggenheim*	2			3		
	Myliobatiformes	Myliobatidae	*Myliobatis goodei*			2			
	Rajiformes	Rajidae	*Amblyraja doellojuradoi*				12		7
			Atlantoraja castelnaui	1					
			Atlantoraja cyclophora				3		

		Atlantoraja platana				2		
		Bathyraja albomaculata						5
		Bathyraja brachyurops						5
		Bathyraja cousseauae						3
		Bathyraja griseocauda						8
		Bathyraja macloviana						4
		Bathyraja magellanica						4
		Bathyraja multispinis						7
		Bathyraja papilionifera						1
		Bathyraja scaphiops				1		5
		*Dipturus argentinensis**					6	1
		Dipturus chilensis					8	
		Psammobatis lentiginosa				6		
		Psammobatis normani				2	1	
		Psammobatis rudis				3	11	4
		Rioraja agassizii	2					
		Sympterygia acuta	2					
		Sympterygia bonapartii					5	
Torpediniformes	Narcinidae	*Discopyge tschudii*					4	
TOTAL			70	9	111	95	144	148
