# Supplementary material for: DNA Barcoding Identifies Argentine Fishes from Marine and Brackish Waters
Source: PLoS One. 2011 Dec 9;6(12):e28655. doi: 10.1371/journal.pone.0028655 (PMC3235135; doi:10.1371/journal.pone.0028655)
Supplement: Table S2 — Details of species and specimens. BOLD specimen numbers given, along with GenBank accession numbers, geographic locality and voucher details. (PDF) [file pone.0028655.s002.pdf]

Identification	Geographic locality	BOLD		GenBank Acc. Nº	Voucher type	
		Sample ID	Process ID			
Acanthistius brasilianus	Argentina, off Patagonian waters	INIDEP-T 0001	FARG001-06	EU074305	Tissue	e-voucher
Acanthistius brasilianus	Argentina, off Patagonian waters	INIDEP-T 0002	FARG002-06	EU074304	Tissue	e-voucher
Acanthistius brasilianus	Argentina, off Patagonian waters	INIDEP-T 0224	FARG224-06	EU074306	Tissue	e-voucher
Acanthistius brasilianus	Argentina, off Patagonian waters	INIDEP-T 0225	FARG225-06	EU074307	Tissue	e-voucher
Acanthistius brasilianus	Argentina, off Patagonian waters	INIDEP-T 0386	FARG387-08		Tissue	e-voucher
Acanthistius brasilianus	Argentina, off Patagonian waters	INIDEP-T 0387	FARG388-08		Tissue	e-voucher
Acanthistius brasilianus	Argentina, off Patagonian waters	INIDEP-T 0388	FARG389-08		Tissue	e-voucher
Acanthistius brasilianus	Argentina, Buenos Aires province coast	INIDEP-T 0522	FARG523-08		Tissue	e-voucher
Allocyttus verrucosus	Argentina, Buenos Aires province coast	INIDEP-T 0543	FARG544-08		Tissue	e-voucher
Allothunnus fallai	Argentina, off Patagonian waters	INIDEP-T 0149	FARG149-06	EU074308	Tissue	e-voucher
Amblyraja doellojuradoi	Argentina, off Patagonian waters	INIDEP-T 0102	FARG102-06	EU074313	Tissue	e-voucher
Amblyraja doellojuradoi	Argentina, off Patagonian waters	INIDEP-T 0241	FARG241-06	EU074310	Tissue	e-voucher
Amblyraja doellojuradoi	Argentina, off Patagonian waters	INIDEP-T 0242	FARG242-06	EU074311	Tissue	e-voucher
Amblyraja doellojuradoi	Argentina, off Patagonian waters	INIDEP-T 0243	FARG243-06	EU074312	Tissue	e-voucher
Amblyraja doellojuradoi	Argentina, off Buenos Aires province	INIDEP-T 0396	FARG397-08		Tissue	e-voucher
Amblyraja doellojuradoi	Argentina, off Buenos Aires province	INIDEP-T 0397	FARG398-08		Tissue	e-voucher
Amblyraja doellojuradoi	Argentina, off Buenos Aires province	INIDEP-T 0399	FARG400-08		Tissue	e-voucher
Amblyraja doellojuradoi	Argentina, off Buenos Aires province	INIDEP-T 0496	FARG497-08		Tissue	e-voucher
Amblyraja doellojuradoi	Argentina, off Buenos Aires province	INIDEP-T 0533	FARG534-08		Tissue	e-voucher
Amblyraja doellojuradoi	Argentina, off Buenos Aires province	INIDEP-T 0534	FARG535-08		Tissue	e-voucher
Amblyraja doellojuradoi	Argentina, off Buenos Aires province	INIDEP-T 0535	FARG536-08		Tissue	e-voucher
Amblyraja doellojuradoi	Argentina, off Buenos Aires province	INIDEP-T 0536	FARG537-08		Tissue	e-voucher
Amblyraja doellojuradoi	Argentina, off Buenos Aires province	INIDEP-T 0537	FARG538-08		Tissue	e-voucher
Amblyraja doellojuradoi	Argentina, off Buenos Aires province	INIDEP-T 0538	FARG539-08		Tissue	e-voucher
Amblyraja sp.	Argentina, off Patagonian waters	INIDEP-T 0130	FARG130-06	EU074317	Tissue	e-voucher
Amblyraja sp.	Argentina, off Patagonian waters	INIDEP-T 0140	FARG140-06	EU074309	Tissue	e-voucher

Amblyraja sp.	Argentina, off Patagonian waters	INIDEP-T 0158	FARG158-06	EU074316	Tissue	e-voucher
Amblyraja sp.	Argentina, off Patagonian waters	INIDEP-T 0171	FARG171-06	EU074315	Tissue	e-voucher
Amblyraja sp.	Argentina, off Patagonian waters	INIDEP-T 0186	FARG186-06	EU074314	Tissue	e-voucher
Anchoa marinii	Argentina, off Buenos Aires province	INIDEP-T 0664	FARG610-09		Tissue	e-voucher
Anchoa marinii	Argentina, off Buenos Aires province	INIDEP-T 0665	FARG611-09		Tissue	e-voucher
Anchoa marinii	Argentina, off Buenos Aires province	INIDEP-T 0666	FARG612-09		Tissue	e-voucher
Atlantoraja castelnaui	Argentina, off Buenos Aires province	INIDEP-T 0406	FARG407-08		Tissue	e-voucher
Atlantoraja cyclophora	Argentina, off Buenos Aires province	INIDEP-T 0469	FARG470-08		Tissue	e-voucher
Atlantoraja cyclophora	Argentina, off Buenos Aires province	INIDEP-T 0471	FARG472-08		Tissue	e-voucher
Atlantoraja cyclophora	Argentina, off Buenos Aires province	INIDEP-T 0474	FARG475-08		Tissue	e-voucher
Atlantoraja platana	Uruguay, common fishing area Argentina-Uruguay	INIDEP-T 0425	FARG426-08		Tissue	e-voucher
Atlantoraja platana	Uruguay, common fishing area Argentina-Uruguay	INIDEP-T 0426	FARG427-08		Tissue	e-voucher
Austrolycus laticinctus	Argentina, off Patagonian waters	INIDEP-T 0125	FARG125-06	EU074319	Tissue	e-voucher
Austrolycus laticinctus	Argentina, off Patagonian waters	INIDEP-T 0134	FARG134-06	EU074320	Tissue	e-voucher
Austrolycus laticinctus	Argentina, off Patagonian waters	INIDEP-T 0315	FARG316-07	EU074318	Tissue	e-voucher
Austrolycus laticinctus	Argentina, off Patagonian waters	INIDEP-T 0382	FARG383-08		Tissue	e-voucher
Bassanago albescens	Argentina, off Patagonian waters	INIDEP-T 0229	FARG229-06	EU074326	Tissue	e-voucher
Bassanago albescens	Argentina, off Patagonian waters	INIDEP-T 0231	FARG231-06	EU074322	Tissue	e-voucher
Bassanago albescens	Argentina, off Patagonian waters	INIDEP-T 0232	FARG232-06	EU074323	Tissue	e-voucher
Bassanago albescens	Argentina, off Patagonian waters	INIDEP-T 0233	FARG233-06	EU074324	Tissue	e-voucher
Bassanago albescens	Argentina, off Patagonian waters	INIDEP-T 0234	FARG234-06	EU074325	Tissue	e-voucher
Bassanago albescens	Argentina, off Buenos Aires province	INIDEP-T 0359	FARG360-07	EU074321	Tissue	e-voucher
Bassanago albescens	Argentina, off Buenos Aires province	INIDEP-T 0400	FARG401-08		Tissue	e-voucher
Bathyraja albomaculata	Argentina, off Patagonian waters	INIDEP-T 0101	FARG101-06	EU074331	Tissue	e-voucher
Bathyraja albomaculata	Argentina, off Patagonian waters	INIDEP-T 0141	FARG141-06	EU074330	Tissue	e-voucher
Bathyraja albomaculata	Argentina, off Patagonian waters	INIDEP-T 0142	FARG142-06	EU074328	Tissue	e-voucher

Bathyraja albomaculata	Argentina, off Patagonian waters	INIDEP-T 0143	FARG143-06	EU074329	Tissue	e-voucher
Bathyraja albomaculata	Argentina, off Patagonian waters	INIDEP-T 0145	FARG145-06	EU074327	Tissue	e-voucher
Bathyraja brachyurops	Argentina, off Patagonian waters	INIDEP-T 0226	FARG226-06	EU074335	Tissue	e-voucher
Bathyraja brachyurops	Argentina, off Patagonian waters	INIDEP-T 0235	FARG235-06	EU074333	Tissue	e-voucher
Bathyraja brachyurops	Argentina, off Patagonian waters	INIDEP-T 0244	FARG244-06	EU074332	Tissue	e-voucher
Bathyraja brachyurops	Argentina, off Patagonian waters	INIDEP-T 0245	FARG245-06	EU074334	Tissue	e-voucher
Bathyraja brachyurops	Argentina, off Buenos Aires province	INIDEP-T 0492	FARG493-08		Tissue	e-voucher
Bathyraja cousseauae	Argentina, off Patagonian waters	INIDEP-T 0172	FARG172-06	EU074338	Tissue	e-voucher
Bathyraja cousseauae	Argentina, off Patagonian waters	INIDEP-T 0181	FARG181-06	EU074337	Tissue	e-voucher
Bathyraja cousseauae	Argentina, off Patagonian waters	INIDEP-T 0185	FARG185-06	EU074336	Tissue	e-voucher
Bathyraja griseocauda	Argentina, off Patagonian waters	INIDEP-T 0153	FARG153-06	EU074345	Tissue	e-voucher
Bathyraja griseocauda	Argentina, off Patagonian waters	INIDEP-T 0155	FARG155-06	EU074346	Tissue	e-voucher
Bathyraja griseocauda	Argentina, off Patagonian waters	INIDEP-T 0156	FARG156-06	EU074340	Tissue	e-voucher
Bathyraja griseocauda	Argentina, off Patagonian waters	INIDEP-T 0161	FARG161-06	EU074339	Tissue	e-voucher
Bathyraja griseocauda	Argentina, off Patagonian waters	INIDEP-T 0258	FARG258-06	EU074342	Tissue	e-voucher
Bathyraja griseocauda	Argentina, off Patagonian waters	INIDEP-T 0259	FARG259-06	EU074341	Tissue	e-voucher
Bathyraja griseocauda	Argentina, off Patagonian waters	INIDEP-T 0260	FARG260-06	EU074343	Tissue	e-voucher
Bathyraja griseocauda	Argentina, off Patagonian waters	INIDEP-T 0261	FARG261-06	EU074344	Tissue	e-voucher
Bathyraja macloviana	Argentina, off Patagonian waters	INIDEP-T 0117	FARG117-06	EU074348	Tissue	e-voucher
Bathyraja macloviana	Argentina, off Patagonian waters	INIDEP-T 0120	FARG120-06	EU074349	Tissue	e-voucher
Bathyraja macloviana	Argentina, off Patagonian waters	INIDEP-T 0121	FARG121-06	EU074347	Tissue	e-voucher
Bathyraja macloviana	Argentina, off Patagonian waters	INIDEP-T 0128	FARG128-06	EU074350	Tissue	e-voucher
Bathyraja magellanica	Argentina, off Patagonian waters	INIDEP-T 0129	FARG129-06	EU074354	Tissue	e-voucher
Bathyraja magellanica	Argentina, off Patagonian waters	INIDEP-T 0147	FARG147-06	EU074353	Tissue	e-voucher
Bathyraja magellanica	Argentina, off Patagonian waters	INIDEP-T 0148	FARG148-06	EU074351	Tissue	e-voucher
Bathyraja magellanica	Argentina, off Patagonian waters	INIDEP-T 0257	FARG257-06	EU074352	Tissue	e-voucher
Bathyraja multispinis	Argentina, off Patagonian waters	INIDEP-T 0146	FARG146-06	EU074357	Tissue	e-voucher
Bathyraja multispinis	Argentina, off Patagonian waters	INIDEP-T 0150	FARG150-06	EU074361	Tissue	e-voucher

Bathyraja multispinis	Argentina, off Patagonian waters	INIDEP-T 0154	FARG154-06	EU074358	Tissue	e-voucher
Bathyraja multispinis	Argentina, off Patagonian waters	INIDEP-T 0262	FARG262-06	EU074360	Tissue	e-voucher
Bathyraja multispinis	Argentina, off Patagonian waters	INIDEP-T 0263	FARG263-06	EU074356	Tissue	e-voucher
Bathyraja multispinis	Argentina, off Patagonian waters	INIDEP-T 0264	FARG264-06	EU074359	Tissue	e-voucher
Bathyraja multispinis	Argentina, off Patagonian waters	INIDEP-T 0266	FARG266-06	EU074355	Tissue	e-voucher
Bathyraja papilionifera	Argentina, off Patagonian waters	INIDEP-T 0500	FARG501-08		Tissue	e-voucher
Bathyraja scaphiops	Argentina, off Patagonian waters	INIDEP-T 0097	FARG097-06	EU074363	Tissue	e-voucher
Bathyraja scaphiops	Argentina, off Patagonian waters	INIDEP-T 0098	FARG098-06	EU074366	Tissue	e-voucher
Bathyraja scaphiops	Argentina, off Patagonian waters	INIDEP-T 0099	FARG099-06	EU074362	Tissue	e-voucher
Bathyraja scaphiops	Argentina, off Patagonian waters	INIDEP-T 0100	FARG100-06	EU074364	Tissue	e-voucher
Bathyraja scaphiops	Argentina, off Patagonian waters	INIDEP-T 0114	FARG114-06	EU074365	Tissue	e-voucher
Bathyraja scaphiops	Argentina, off Buenos Aires province	INIDEP-T 0466	FARG467-08		Tissue	e-voucher
Bovichtus chilensis	Argentina, Buenos Aires province coast	INIDEP-T 0563	FARG559-09		Tissue	e-voucher
Bovichtus chilensis	Argentina, Buenos Aires province coast	INIDEP-T 0564	FARG560-09		Tissue	e-voucher
Bovichtus chilensis	Argentina, Buenos Aires province coast	INIDEP-T 0682	FARG628-09		Tissue	e-voucher
Bovichtus chilensis	Argentina, Buenos Aires province coast	INIDEP-T 0693	FARG639-09		Tissue	e-voucher
Brama brama	Argentina, off Patagonian waters	INIDEP-T 0008	FARG008-06	EU074367	Tissue	e-voucher
Brama brama	Argentina, off Buenos Aires province	INIDEP-T 0432	FARG433-08		Tissue	e-voucher
Brama brama	Argentina, off Buenos Aires province	INIDEP-T 0435	FARG436-08		Tissue	e-voucher
Brama brama	Argentina, off Buenos Aires province	INIDEP-T 0439	FARG440-08		Tissue	e-voucher
Brama brama	Argentina, off Buenos Aires province	INIDEP-T 0503	FARG504-08		Tissue	e-voucher
Brevoortia aurea	Argentina, Mar Chiquita coastal lagoon	INIDEP-T 0270b	FARG271-07	EU074372	Tissue	e-voucher
Brevoortia aurea	Argentina, Mar Chiquita coastal lagoon	INIDEP-T 0271	FARG272-07	EU074371	Tissue	e-voucher
Brevoortia aurea	Argentina, Mar Chiquita coastal lagoon	INIDEP-T 0272	FARG273-07	EU074370	Tissue	e-voucher
Brevoortia aurea	Argentina, Mar Chiquita coastal lagoon	INIDEP-T 0273	FARG274-07	EU074369	Tissue	e-voucher
Brevoortia aurea	Argentina, Mar Chiquita coastal lagoon	INIDEP-T 0291	FARG292-07	EU074368	Tissue	e-voucher
Brevoortia aurea	Argentina, Mar Chiquita coastal lagoon	INIDEP-T 0646	FARG592-09		Tissue	e-voucher
Brevoortia aurea	Argentina, Mar Chiquita coastal lagoon	INIDEP-T 0647	FARG593-09		Tissue	e-voucher

Brevoortia aurea	Argentina, Mar Chiquita coastal lagoon	INIDEP-T 0648	FARG594-09		Tissue	e-voucher
Brevoortia aurea	Argentina, Mar Chiquita coastal lagoon	INIDEP-T 0649	FARG595-09		Tissue	e-voucher
Brevoortia aurea	Argentina, Mar Chiquita coastal lagoon	INIDEP-T 0677	FARG623-09		Tissue	e-voucher
Brevoortia aurea	Argentina, Mar Chiquita coastal lagoon	INIDEP-T 0678	FARG624-09		Tissue	e-voucher
Brevoortia aurea	Argentina, Mar Chiquita coastal lagoon	INIDEP-T 0679	FARG625-09		Tissue	e-voucher
Brevoortia aurea	Argentina, Mar Chiquita coastal lagoon	INIDEP-T 0680	FARG626-09		Tissue	e-voucher
Brevoortia aurea	Argentina, Mar Chiquita coastal lagoon	INIDEP-T 0681	FARG627-09		Tissue	e-voucher
Brevoortia aurea	Argentina, Mar Chiquita coastal lagoon	INIDEP-T 0685	FARG631-09		Tissue	e-voucher
Callorhinchus callorynchus	Argentina, off Patagonian waters	INIDEP-T 0003	FARG003-06	EU074379	Tissue	e-voucher
Callorhinchus callorynchus	Argentina, off Patagonian waters	INIDEP-T 0004	FARG004-06	EU074378	Tissue	e-voucher
Callorhinchus callorynchus	Argentina, off Patagonian waters	INIDEP-T 0006	FARG006-06	EU074375	Tissue	e-voucher
Callorhinchus callorynchus	Argentina, off Patagonian waters	INIDEP-T 0007	FARG007-06	EU074381	Tissue	e-voucher
Callorhinchus callorynchus	Argentina, off Patagonian waters	INIDEP-T 0223	FARG223-06	EU074380	Tissue	e-voucher
Callorhinchus callorynchus	Argentina, Mar Chiquita coastal lagoon	INIDEP-T 0329	FARG330-07	EU074377	Tissue	e-voucher
Callorhinchus callorynchus	Argentina, off Patagonian waters	INIDEP-T 0379	FARG380-08		Tissue	e-voucher
Callorhinchus callorynchus	Argentina, off Patagonian waters	INIDEP-T 0380	FARG381-08		Tissue	e-voucher
Callorhinchus callorynchus	Argentina, off Patagonian waters	INIDEP-T 0381	FARG382-08		Tissue	e-voucher
Cnesterodon decemmaculatus	Argentina, Parque Lago lagoon, Buenos Aires	INIDEP-T 0671	FARG617-09		Tissue	e-voucher
Coelorinchus fasciatus	Argentina, off Patagonian waters	INIDEP-T 0163	FARG163-06	EU074374	Tissue	e-voucher
Coelorinchus fasciatus	Argentina, off Patagonian waters	INIDEP-T 0165	FARG165-06	EU074373	Tissue	e-voucher
Coelorinchus marinii	Argentina, off Buenos Aires province	INIDEP-T 0353	FARG354-07	EU074385	Tissue	e-voucher
Coelorinchus marinii	Argentina, off Buenos Aires province	INIDEP-T 0354	FARG355-07	EU074384	Tissue	e-voucher
Coelorinchus marinii	Argentina, off Buenos Aires province	INIDEP-T 0355	FARG356-07	EU074383	Tissue	e-voucher
Coelorinchus marinii	Argentina, off Buenos Aires province	INIDEP-T 0356	FARG357-07	EU074382	Tissue	e-voucher
Coelorinchus marinii	Argentina, off Buenos Aires province	INIDEP-T 0460	FARG461-08		Tissue	e-voucher
Coelorinchus marinii	Argentina, off Buenos Aires province	INIDEP-T 0461	FARG462-08		Tissue	e-voucher
Conger orbignianus	Argentina, off Buenos Aires province	INIDEP-T 0430	FARG431-08		Tissue	e-voucher

Conger orbignianus	Argentina, off Buenos Aires province	INIDEP-T 0499	FARG500-08		Tissue	e-voucher
Conger orbignianus	Argentina, Buenos Aires province coast	INIDEP-T 0641	FARG587-09		Tissue	e-voucher
Conger orbignianus	Argentina, Buenos Aires province coast	INIDEP-T 0642	FARG588-09		Tissue	e-voucher
Conger orbignianus	Argentina, Buenos Aires province coast	INIDEP-T 0697	FARG643-09		Tissue	e-voucher
Congiopodus peruvianus	Argentina, off Patagonian waters	INIDEP-T 0009	FARG009-06	EU074388	Tissue	e-voucher
Congiopodus peruvianus	Argentina, off Patagonian waters	INIDEP-T 0010	FARG010-06	EU074390	Tissue	e-voucher
Congiopodus peruvianus	Argentina, off Patagonian waters	INIDEP-T 0011	FARG011-06	EU074389	Tissue	e-voucher
Congiopodus peruvianus	Argentina, off Patagonian waters	INIDEP-T 0012	FARG012-06	EU074386	Tissue	e-voucher
Congiopodus peruvianus	Argentina, off Patagonian waters	INIDEP-T 0013	FARG013-06	EU074387	Tissue	e-voucher
Congiopodus peruvianus	Argentina, Buenos Aires province coast	INIDEP-T 0637	FARG583-09		Tissue	e-voucher
Congiopodus peruvianus	Argentina, Buenos Aires province coast	INIDEP-T 0695	FARG641-09		Tissue	e-voucher
Corydoras paleatus	Argentina, Parque Lago lagoon, Buenos Aires	INIDEP-T 0672	FARG618-09		Tissue	e-voucher
Corydoras paleatus	Argentina, Parque Lago lagoon, Buenos Aires	INIDEP-T 0673	FARG619-09		Tissue	e-voucher
Cottoperca gobio	Argentina, off Patagonian waters	INIDEP-T 0116	FARG116-06	EU074394	Tissue	e-voucher
Cottoperca gobio	Argentina, off Patagonian waters	INIDEP-T 0118	FARG118-06	EU074391	Tissue	e-voucher
Cottoperca gobio	Argentina, off Patagonian waters	INIDEP-T 0124	FARG124-06	EU074392	Tissue	e-voucher
Cottoperca gobio	Argentina, off Patagonian waters	INIDEP-T 0222	FARG222-06	EU074393	Tissue	e-voucher
Cynoscion guatucupa	Argentina, Mar Chiquita coastal lagoon	INIDEP-T 0286	FARG287-07	EU074399	Tissue	e-voucher
Cynoscion guatucupa	Argentina, Mar Chiquita coastal lagoon	INIDEP-T 0304	FARG305-07	EU074398	Tissue	e-voucher
Cynoscion guatucupa	Argentina, Mar Chiquita coastal lagoon	INIDEP-T 0305	FARG306-07	EU074397	Tissue	e-voucher
Cynoscion guatucupa	Argentina, Mar Chiquita coastal lagoon	INIDEP-T 0306	FARG307-07	EU074396	Tissue	e-voucher
Cynoscion guatucupa	Argentina, Mar Chiquita coastal lagoon	INIDEP-T 0307	FARG308-07	EU074395	Tissue	e-voucher
Dactylopterus volitans	Argentina, Buenos Aires province coast	INIDEP-T 0541	FARG542-08		Tissue	e-voucher
Dipturus argentinensis	Argentina, off Patagonian waters	INIDEP-T 0019	FARG019-06	EU074405	Tissue	Specimen
Dipturus argentinensis	Argentina, off Patagonian waters	INIDEP-T 0020	FARG020-06	EU074409	Tissue	Specimen
Dipturus argentinensis	Argentina, off Patagonian waters	INIDEP-T 0021	FARG021-06	EU074410	Tissue	Specimen

Dipturus argentinensis	Argentina, off Patagonian waters	INIDEP-T 0127	FARG127-06	EU074411	Tissue	Specimen
Dipturus argentinensis	Argentina, off Patagonian waters	INIDEP-T 0317	FARG318-07	EU074408	Tissue	Specimen
Dipturus argentinensis	Argentina, off Patagonian waters	INIDEP-T 0318	FARG319-07	EU074407	Tissue	Specimen
Dipturus argentinensis	Argentina, off Patagonian waters	INIDEP-T 0319	FARG320-07	EU074406	Tissue	Specimen
Dipturus chilensis	Argentina, off Patagonian waters	INIDEP-T 0014	FARG014-06	EU074404	Tissue	e-voucher
Dipturus chilensis	Argentina, off Patagonian waters	INIDEP-T 0015	FARG015-06	EU074402	Tissue	e-voucher
Dipturus chilensis	Argentina, off Patagonian waters	INIDEP-T 0016	FARG016-06	EU074400	Tissue	e-voucher
Dipturus chilensis	Argentina, off Patagonian waters	INIDEP-T 0018	FARG018-06	EU074403	Tissue	e-voucher
Dipturus chilensis	Uruguay, common fishing area Argentina-Uruguay	INIDEP-T 0337	FARG338-07	EU074401	Tissue	e-voucher
Dipturus chilensis	Argentina, off Buenos Aires province	INIDEP-T 0592	FARG567-09		Tissue	e-voucher
Dipturus chilensis	Argentina, off Buenos Aires province	INIDEP-T 0593	FARG568-09		Tissue	e-voucher
Dipturus chilensis	Argentina, off Buenos Aires province	INIDEP-T 0594	FARG569-09		Tissue	e-voucher
Discopyge tschudii	Argentina, off Patagonian waters	INIDEP-T 0022	FARG022-06	EU074415	Tissue	e-voucher
Discopyge tschudii	Argentina, off Patagonian waters	INIDEP-T 0023	FARG023-06	EU074412	Tissue	e-voucher
Discopyge tschudii	Argentina, off Patagonian waters	INIDEP-T 0024	FARG024-06	EU074414	Tissue	e-voucher
Discopyge tschudii	Argentina, off Patagonian waters	INIDEP-T 0251	FARG251-06	EU074413	Tissue	e-voucher
Dissostichus eleginoides	Argentina, off Patagonian waters	INIDEP-T 0159	FARG159-06	EU074418	Tissue	e-voucher
Dissostichus eleginoides	Argentina, off Patagonian waters	INIDEP-T 0160	FARG160-06	EU074420	Tissue	e-voucher
Dissostichus eleginoides	Argentina, off Patagonian waters	INIDEP-T 0170	FARG170-06	EU074419	Tissue	e-voucher
Dissostichus eleginoides	Argentina, off Patagonian waters	INIDEP-T 0277	FARG278-07	EU074417	Tissue	e-voucher
Dissostichus eleginoides	Argentina, off Patagonian waters	INIDEP-T 0278	FARG279-07	EU074416	Tissue	e-voucher
Dissostichus eleginoides	Argentina, off Buenos Aires province	INIDEP-T 0487	FARG488-08		Tissue	e-voucher
Eleginops maclovinus	Argentina, off Patagonian waters	INIDEP-T 0025	FARG025-06	EU074421	Tissue	e-voucher
Eleginops maclovinus	Argentina, off Patagonian waters	INIDEP-T 0383	FARG384-08		Tissue	e-voucher
Eleginops maclovinus	Argentina, off Patagonian waters	INIDEP-T 0384	FARG385-08		Tissue	e-voucher
Eleginops maclovinus	Argentina, off Patagonian waters	INIDEP-T 0385	FARG386-08		Tissue	e-voucher
Eleginops maclovinus	Argentina, Buenos Aires province coast	INIDEP-T 0511	FARG512-08		Tissue	e-voucher

Eleginops maclovinus	Argentina, Buenos Aires province coast	INIDEP-T 0512	FARG513-08		Tissue	e-voucher
Eleginops maclovinus	Argentina, Buenos Aires province coast	INIDEP-T 0513	FARG514-08		Tissue	e-voucher
Engraulis anchoita	Argentina, off Patagonian waters	INIDEP-T 0026	FARG026-06	EU074422	Tissue	e-voucher
Engraulis anchoita	Argentina, off Patagonian waters	INIDEP-T 0027	FARG027-06	EU074424	Tissue	e-voucher
Engraulis anchoita	Argentina, off Patagonian waters	INIDEP-T 0029	FARG029-06	EU074423	Tissue	e-voucher
Galeorhinus galeus	Argentina, coastal Patagonian waters	INIDEP-T 0039	FARG039-06	EU074425	Tissue	e-voucher
Genypterus blacodes	Argentina, off Patagonian waters	INIDEP-T 0032	FARG032-06	EU074428	Tissue	e-voucher
Genypterus blacodes	Argentina, off Patagonian waters	INIDEP-T 0033	FARG033-06	EU074430	Tissue	e-voucher
Genypterus blacodes	Argentina, off Patagonian waters	INIDEP-T 0034	FARG034-06	EU074427	Tissue	e-voucher
Genypterus blacodes	Argentina, off Patagonian waters	INIDEP-T 0035	FARG035-06	EU074426	Tissue	e-voucher
Genypterus blacodes	Argentina, off Patagonian waters	INIDEP-T 0036	FARG036-06	EU074429	Tissue	e-voucher
Genypterus brasiliensis	Argentina, off Patagonian waters	INIDEP-T 0037	FARG037-06	EU074431	Tissue	e-voucher
Genypterus brasiliensis	Argentina, off Patagonian waters	INIDEP-T 0038	FARG038-06	EU074432	Tissue	e-voucher
Gobiosoma parri	Argentina, Mar Chiquita coastal lagoon	INIDEP-T 0688	FARG634-09		Tissue	e-voucher
Gobiosoma parri	Argentina, Mar Chiquita coastal lagoon	INIDEP-T 0689	FARG635-09		Tissue	e-voucher
Gobiosoma parri	Argentina, Mar Chiquita coastal lagoon	INIDEP-T 0690	FARG636-09		Tissue	e-voucher
Gobiosoma parri	Argentina, Mar Chiquita coastal lagoon	INIDEP-T 0691	FARG637-09		Tissue	e-voucher
Gobiosoma parri	Argentina, Mar Chiquita coastal lagoon	INIDEP-T 0692	FARG638-09		Tissue	e-voucher
Gymnoscopelus nicholsi	Argentina, off Patagonian waters	INIDEP-T 0168	FARG168-06	EU074435	Tissue	e-voucher
Gymnoscopelus nicholsi	Argentina, off Patagonian waters	INIDEP-T 0169	FARG169-06	EU074439	Tissue	e-voucher
Gymnoscopelus nicholsi	Argentina, off Patagonian waters	INIDEP-T 0174	FARG174-06	EU074436	Tissue	e-voucher
Gymnoscopelus nicholsi	Argentina, off Patagonian waters	INIDEP-T 0175	FARG175-06	EU074438	Tissue	e-voucher
Gymnoscopelus nicholsi	Argentina, off Patagonian waters	INIDEP-T 0176	FARG176-06	EU074434	Tissue	e-voucher
Gymnoscopelus nicholsi	Argentina, off Patagonian waters	INIDEP-T 0177	FARG177-06	EU074437	Tissue	e-voucher
Gymnoscopelus nicholsi	Argentina, off Patagonian waters	INIDEP-T 0180	FARG180-06	EU074433	Tissue	e-voucher
Gymnoscopelus nicholsi	Argentina, off Patagonian waters	INIDEP-T 0187	FARG187-06	EU074440	Tissue	e-voucher
Gymnoscopelus piabilis	Argentina, off Patagonian waters	INIDEP-T 0188	FARG188-06	EU074441	Tissue	e-voucher
Helicolenus lahillei	Argentina, off Buenos Aires province	INIDEP-T 0454	FARG455-08		Tissue	e-voucher

Helicolenus lahillei	Argentina, off Buenos Aires province	INIDEP-T 0455	FARG456-08		Tissue	e-voucher
Helicolenus lahillei	Argentina, off Buenos Aires province	INIDEP-T 0456	FARG457-08		Tissue	e-voucher
Helicolenus lahillei	Argentina, Buenos Aires province coast	INIDEP-T 0595	FARG570-09		Tissue	e-voucher
Helicolenus lahillei	Argentina, Buenos Aires province coast	INIDEP-T 0596	FARG571-09		Tissue	e-voucher
Helicolenus lahillei	Argentina, Buenos Aires province coast	INIDEP-T 0597	FARG572-09		Tissue	e-voucher
Helicolenus lahillei	Argentina, Buenos Aires province coast	INIDEP-T 0598	FARG573-09		Tissue	e-voucher
Helicolenus lahillei	Argentina, Buenos Aires province coast	INIDEP-T 0599	FARG574-09		Tissue	e-voucher
Helicolenus lahillei	Argentina, Buenos Aires province coast	INIDEP-T 0600	FARG575-09		Tissue	e-voucher
Helicolenus lahillei	Argentina, Buenos Aires province coast	INIDEP-T 0601	FARG576-09		Tissue	e-voucher
Helicolenus lahillei	Argentina, Buenos Aires province coast	INIDEP-T 0602	FARG577-09		Tissue	e-voucher
Helicolenus lahillei	Argentina, Buenos Aires province coast	INIDEP-T 0603	FARG578-09		Tissue	e-voucher
Helicolenus lahillei	Argentina, Buenos Aires province coast	INIDEP-T 0628	FARG579-09		Tissue	e-voucher
Hippocampus patagonicus	Argentina, San Antonio Oeste coast	INIDEP-T 0505	FARG506-08		Tissue	e-voucher
Hippocampus patagonicus	Argentina, San Antonio Oeste coast	INIDEP-T 0506	FARG507-08		Tissue	e-voucher
Hippocampus patagonicus	Argentina, San Antonio Oeste coast	INIDEP-T 0507	FARG508-08		Tissue	e-voucher
Hippocampus patagonicus	Argentina, San Antonio Oeste coast	INIDEP-T 0508	FARG509-08		Tissue	e-voucher
Hippocampus patagonicus	Argentina, San Antonio Oeste coast	INIDEP-T 0509	FARG510-08		Tissue	e-voucher
Hippocampus patagonicus	Argentina, San Antonio Oeste coast	INIDEP-T 0510	FARG511-08		Tissue	e-voucher
Hypleurochilus fissicornis	Argentina, Buenos Aires province coast	INIDEP-T 0565	FARG561-09		Tissue	e-voucher
Icichthys australis	Argentina, off Patagonian waters	INIDEP-T 0539	FARG540-08		Tissue	e-voucher
Iluocoetes fimbriatus	Argentina, off Buenos Aires province	INIDEP-T 0040	FARG040-06	EU074442	Tissue	e-voucher
Iluocoetes fimbriatus	Argentina, off Buenos Aires province	INIDEP-T 0237	FARG237-06	EU074446	Tissue	e-voucher
Iluocoetes fimbriatus	Argentina, off Buenos Aires province	INIDEP-T 0238	FARG238-06	EU074443	Tissue	e-voucher
Iluocoetes fimbriatus	Argentina, off Patagonian waters	INIDEP-T 0239	FARG239-06	EU074444	Tissue	e-voucher
Iluocoetes fimbriatus	Argentina, off Patagonian waters	INIDEP-T 0240	FARG240-06	EU074445	Tissue	e-voucher
Iluocoetes fimbriatus	Argentina, off Patagonian waters	INIDEP-T 0368	FARG369-08		Tissue	e-voucher
Iluocoetes fimbriatus	Argentina, off Patagonian waters	INIDEP-T 0369	FARG370-08		Tissue	e-voucher
Iluocoetes fimbriatus	Argentina, off Patagonian waters	INIDEP-T 0370	FARG371-08		Tissue	e-voucher

Iluocoetes fimbriatus	Argentina, off Buenos Aires province	INIDEP-T 0554	FARG555-09		Tissue	e-voucher
Iluocoetes fimbriatus	Argentina, off Buenos Aires province	INIDEP-T 0555	FARG556-09		Tissue	e-voucher
Iluocoetes fimbriatus	Argentina, off Buenos Aires province	INIDEP-T 0556	FARG557-09		Tissue	e-voucher
Jenynsia multidentata	Argentina, Mar Chiquita coastal lagoon	INIDEP-T 0409	FARG410-08		Tissue	e-voucher
Jenynsia multidentata	Argentina, Mar Chiquita coastal lagoon	INIDEP-T 0410	FARG411-08		Tissue	e-voucher
Jenynsia multidentata	Argentina, Mar Chiquita coastal lagoon	INIDEP-T 0427	FARG428-08		Tissue	e-voucher
Jenynsia multidentata	Argentina, Mar Chiquita coastal lagoon	INIDEP-T 0428	FARG429-08		Tissue	e-voucher
Jenynsia multidentata	Argentina, Mar Chiquita coastal lagoon	INIDEP-T 0515	FARG516-08		Tissue	e-voucher
Jenynsia multidentata	Argentina, Mar Chiquita coastal lagoon	INIDEP-T 0516	FARG517-08		Tissue	e-voucher
Jenynsia multidentata	Argentina, Parque Lago lagoon, Buenos Aires	INIDEP-T 0667	FARG613-09		Tissue	e-voucher
Jenynsia multidentata	Argentina, Parque Lago lagoon, Buenos Aires	INIDEP-T 0668	FARG614-09		Tissue	e-voucher
Jenynsia multidentata	Argentina, Parque Lago lagoon, Buenos Aires	INIDEP-T 0669	FARG615-09		Tissue	e-voucher
Jenynsia multidentata	Argentina, Parque Lago lagoon, Buenos Aires	INIDEP-T 0670	FARG616-09		Tissue	e-voucher
Lopholatilus villarii	Uruguay, Common fishing area Argentina-Uruguay	INIDEP-T 0338	FARG339-07	EU074447	Tissue	e-voucher
Lycengraulis grossidens	Argentina, Mar Chiquita coastal lagoon	INIDEP-T 0279	FARG280-07	EU074449	Tissue	e-voucher
Lycengraulis grossidens	Argentina, Mar Chiquita coastal lagoon	INIDEP-T 0281	FARG282-07	EU074448	Tissue	e-voucher
Lycengraulis grossidens	Argentina, Mar Chiquita coastal lagoon	INIDEP-T 0517	FARG518-08		Tissue	e-voucher
Lycengraulis grossidens	Argentina, Mar Chiquita coastal lagoon	INIDEP-T 0518	FARG519-08		Tissue	e-voucher
Lycengraulis grossidens	Argentina, Mar Chiquita coastal lagoon	INIDEP-T 0519	FARG520-08		Tissue	e-voucher
Lycengraulis grossidens	Argentina, Mar Chiquita coastal lagoon	INIDEP-T 0520	FARG521-08		Tissue	e-voucher
Lycengraulis grossidens	Argentina, Mar Chiquita coastal lagoon	INIDEP-T 0521	FARG522-08		Tissue	e-voucher
Macrourus carinatus	Argentina, off Patagonian waters	INIDEP-T 0106	FARG106-06	EU074450	Tissue	e-voucher
Macrourus carinatus	Argentina, off Patagonian waters	INIDEP-T 0107	FARG107-06	EU074453	Tissue	e-voucher

Macrourus carinatus	Argentina, off Patagonian waters	INIDEP-T 0108	FARG108-06	EU074454	Tissue	e-voucher
Macrourus carinatus	Argentina, off Patagonian waters	INIDEP-T 0109	FARG109-06	EU074451	Tissue	e-voucher
Macrourus carinatus	Argentina, off Patagonian waters	INIDEP-T 0110	FARG110-06	EU074455	Tissue	e-voucher
Macrourus carinatus	Argentina, off Patagonian waters	INIDEP-T 0236	FARG236-06	EU074452	Tissue	e-voucher
Macruronus magellanicus	Argentina, off Patagonian waters	INIDEP-T 0041	FARG041-06	EU074456	Tissue	e-voucher
Macruronus magellanicus	Argentina, off Patagonian waters	INIDEP-T 0042	FARG042-06	EU074460	Tissue	e-voucher
Macruronus magellanicus	Argentina, off Patagonian waters	INIDEP-T 0043	FARG043-06	EU074458	Tissue	e-voucher
Macruronus magellanicus	Argentina, off Patagonian waters	INIDEP-T 0044	FARG044-06	EU074457	Tissue	e-voucher
Macruronus magellanicus	Argentina, off Patagonian waters	INIDEP-T 0045	FARG045-06	EU074459	Tissue	e-voucher
Mancopsetta maculata	Argentina, off Patagonian waters	INIDEP-T 0115	FARG115-06	EU074462	Tissue	e-voucher
Mancopsetta maculata	Argentina, off Patagonian waters	INIDEP-T 0183	FARG183-06	EU074461	Tissue	e-voucher
Menticirrhus americanus	Argentina, off Buenos Aires province	INIDEP-T 0312	FARG313-07	EU074467	Tissue	e-voucher
Menticirrhus americanus	Argentina, off Buenos Aires province	INIDEP-T 0313	FARG314-07	EU074466	Tissue	e-voucher
Menticirrhus americanus	Argentina, Mar Chiquita coastal lagoon	INIDEP-T 0323	FARG324-07	EU074465	Tissue	e-voucher
Menticirrhus americanus	Argentina, Mar Chiquita coastal lagoon	INIDEP-T 0324	FARG325-07	EU074464	Tissue	e-voucher
Menticirrhus americanus	Argentina, Mar Chiquita coastal lagoon	INIDEP-T 0325	FARG326-07	EU074463	Tissue	e-voucher
Merluccius australis	Argentina, off Patagonian waters	INIDEP-T 0173	FARG173-06	EU074468	Tissue	e-voucher
Merluccius australis	New Zealand	INIDEP-T 0445	FARG446-08		Tissue	e-voucher
Merluccius australis	New Zealand	INIDEP-T 0446	FARG447-08		Tissue	e-voucher
Merluccius australis	New Zealand	INIDEP-T 0447	FARG448-08		Tissue	e-voucher
Merluccius australis	New Zealand	INIDEP-T 0449	FARG450-08		Tissue	e-voucher
Merluccius australis	New Zealand	INIDEP-T 0450	FARG451-08		Tissue	e-voucher
Merluccius australis	Argentina, off Patagonian waters	INIDEP-T 0476	FARG477-08		Tissue	e-voucher
Merluccius hubbsi	Argentina, off Patagonian waters	INIDEP-T 0046	FARG046-06	EU074471	Tissue	e-voucher
Merluccius hubbsi	Argentina, off Patagonian waters	INIDEP-T 0047	FARG047-06	EU074470	Tissue	e-voucher
Merluccius hubbsi	Argentina, off Patagonian waters	INIDEP-T 0048	FARG048-06	EU074476	Tissue	e-voucher
Merluccius hubbsi	Argentina, off Patagonian waters	INIDEP-T 0049	FARG049-06	EU074477	Tissue	e-voucher
Merluccius hubbsi	Argentina, off Patagonian waters	INIDEP-T 0050	FARG050-06	EU074478	Tissue	e-voucher

Merluccius hubbsi	Argentina, off Patagonian waters	INIDEP-T 0246	FARG246-06	EU074474	Tissue	e-voucher
Merluccius hubbsi	Argentina, off Patagonian waters	INIDEP-T 0247	FARG247-06	EU074469	Tissue	e-voucher
Merluccius hubbsi	Argentina, off Patagonian waters	INIDEP-T 0248	FARG248-06	EU074472	Tissue	e-voucher
Merluccius hubbsi	Argentina, off Patagonian waters	INIDEP-T 0249	FARG249-06	EU074473	Tissue	e-voucher
Merluccius hubbsi	Argentina, off Patagonian waters	INIDEP-T 0250	FARG250-06	EU074475	Tissue	e-voucher
Micromesistius australis	Argentina, off Patagonian waters	INIDEP-T 0103	FARG103-06	EU074480	Tissue	e-voucher
Micromesistius australis	Argentina, off Patagonian waters	INIDEP-T 0104	FARG104-06	EU074479	Tissue	e-voucher
Micromesistius australis	Argentina, off Patagonian waters	INIDEP-T 0105	FARG105-06	EU074481	Tissue	e-voucher
Micromesistius australis	Argentina, off Patagonian waters	INIDEP-T 0367	FARG368-08		Tissue	e-voucher
Micromesistius australis	Argentina, off Patagonian waters	INIDEP-T 0371	FARG372-08		Tissue	e-voucher
Micromesistius australis	Argentina, off Patagonian waters	INIDEP-T 0372	FARG373-08		Tissue	e-voucher
Micromesistius australis	Argentina, off Buenos Aires province	INIDEP-T 0489	FARG490-08		Tissue	e-voucher
Micropogonias furnieri	Argentina, Mar Chiquita coastal lagoon	INIDEP-T 0288	FARG289-07	EU074482	Tissue	e-voucher
Micropogonias furnieri	Argentina, Mar Chiquita coastal lagoon	INIDEP-T 0411	FARG412-08		Tissue	e-voucher
Micropogonias furnieri	Argentina, Mar Chiquita coastal lagoon	INIDEP-T 0412	FARG413-08		Tissue	e-voucher
Micropogonias furnieri	Argentina, Mar Chiquita coastal lagoon	INIDEP-T 0413	FARG414-08		Tissue	e-voucher
Micropogonias furnieri	Argentina, Mar Chiquita coastal lagoon	INIDEP-T 0414	FARG415-08		Tissue	e-voucher
Micropogonias furnieri	Argentina, Mar Chiquita coastal lagoon	INIDEP-T 0415	FARG416-08		Tissue	e-voucher
Micropogonias furnieri	Argentina, Mar Chiquita coastal lagoon	INIDEP-T 0654	FARG600-09		Tissue	e-voucher
Micropogonias furnieri	Argentina, Mar Chiquita coastal lagoon	INIDEP-T 0684	FARG630-09		Tissue	e-voucher
Mugil platanus	Argentina, Mar Chiquita coastal lagoon	INIDEP-T 0293	FARG294-07	EU074485	Tissue	e-voucher
Mugil platanus	Argentina, Mar Chiquita coastal lagoon	INIDEP-T 0297	FARG298-07	EU074484	Tissue	e-voucher
Mugil platanus	Argentina, Mar Chiquita coastal lagoon	INIDEP-T 0330	FARG331-07	EU074483	Tissue	e-voucher
Mugil platanus	Argentina, Mar Chiquita coastal lagoon	INIDEP-T 0422	FARG423-08		Tissue	e-voucher
Mugil platanus	Argentina, Mar Chiquita coastal lagoon	INIDEP-T 0452	FARG453-08		Tissue	e-voucher
Mugil platanus	Argentina, Mar Chiquita coastal lagoon	INIDEP-T 0683	FARG629-09		Tissue	e-voucher
Mullus argentinae	Argentina, Buenos Aires province coast	INIDEP-T 0544	FARG545-08		Tissue	e-voucher
Mullus argentinae	Argentina, Buenos Aires province coast	INIDEP-T 0545	FARG546-08		Tissue	e-voucher

Mullus argentinae	Argentina, Buenos Aires province coast	INIDEP-T 0546	FARG547-08		Tissue	e-voucher
Mullus argentinae	Argentina, Buenos Aires province coast	INIDEP-T 0547	FARG548-08		Tissue	e-voucher
Mullus argentinae	Argentina, Buenos Aires province coast	INIDEP-T 0548	FARG549-08		Tissue	e-voucher
Mustelus schmitti	Argentina, off Patagonian waters	INIDEP-T 0051	FARG051-06	EU074488	Tissue	e-voucher
Mustelus schmitti	Argentina, off Patagonian waters	INIDEP-T 0052	FARG052-06	EU074487	Tissue	e-voucher
Mustelus schmitti	Uruguay, Common fishing area Argentina-Uruguay	INIDEP-T 0331	FARG332-07	EU074486	Tissue	e-voucher
Myliobatis goodei	Argentina, Mar Chiquita coastal lagoon	INIDEP-T 0322	FARG323-07	EU074489	Tissue	e-voucher
Myliobatis goodei	Argentina, Mar Chiquita coastal lagoon	INIDEP-T 0451	FARG452-08		Tissue	e-voucher
Myxine australis	Argentina, off Patagonian waters	INIDEP-T 0132	FARG132-06	EU074490	Tissue	e-voucher
Nemadactylus bergi	Argentina, off Patagonian waters	INIDEP-T 0053	FARG053-06	EU074496	Tissue	e-voucher
Nemadactylus bergi	Argentina, coastal Patagonian waters	INIDEP-T 0054	FARG054-06	EU074497	Tissue	e-voucher
Nemadactylus bergi	Argentina, coastal Patagonian waters	INIDEP-T 0055	FARG055-06	EU074493	Tissue	e-voucher
Nemadactylus bergi	Argentina, coastal Patagonian waters	INIDEP-T 0056	FARG056-06	EU074495	Tissue	e-voucher
Nemadactylus bergi	Argentina, coastal Patagonian waters	INIDEP-T 0057	FARG057-06	EU074494	Tissue	e-voucher
Nemadactylus bergi	Argentina, off La Plata River	INIDEP-T 0345	FARG346-07	EU074492	Tissue	e-voucher
Nemadactylus bergi	Argentina, off La Plata River	INIDEP-T 0346	FARG347-07	EU074491	Tissue	e-voucher
Neoachiropsetta milfordi	Argentina, off Patagonian waters	INIDEP-T 0157	FARG157-06	EU074499	Tissue	e-voucher
Neoachiropsetta milfordi	Argentina, off Patagonian waters	INIDEP-T 0182	FARG182-06	EU074498	Tissue	e-voucher
Notomyxine tridentiger	Argentina, off Patagonian waters	INIDEP-T 0131	FARG131-06	EU074500	Tissue	e-voucher
Notophycis marginata	Argentina, off Patagonian waters	INIDEP-T 0111	FARG111-06	EU074501	Tissue	e-voucher
Notophycis marginata	Argentina, off Patagonian waters	INIDEP-T 0112	FARG112-06	EU074504	Tissue	e-voucher
Notophycis marginata	Argentina, off Patagonian waters	INIDEP-T 0113	FARG113-06	EU074502	Tissue	e-voucher
Notophycis marginata	Argentina, off Patagonian waters	INIDEP-T 0166	FARG166-06	EU074503	Tissue	e-voucher
Notopogon fernandezianus	Argentina, off La Plata River	INIDEP-T 0342	FARG343-07	EU074506	Tissue	e-voucher
Notopogon fernandezianus	Argentina, off La Plata River	INIDEP-T 0343	FARG344-07	EU074505	Tissue	e-voucher
Notorynchus cepedianus	Argentina, off Patagonian waters	INIDEP-T 0230	FARG230-06	EU074507	Tissue	e-voucher
Odontesthes argentinensis	Argentina, Mar Chiquita coastal lagoon	INIDEP-T 0274	FARG275-07	EU074512	Tissue	e-voucher

Odontesthes argentinensis	Argentina, Mar Chiquita coastal lagoon	INIDEP-T 0275	FARG276-07	EU074511	Tissue	e-voucher
Odontesthes argentinensis	Argentina, Mar Chiquita coastal lagoon	INIDEP-T 0294	FARG295-07	EU074510	Tissue	e-voucher
Odontesthes argentinensis	Argentina, Mar Chiquita coastal lagoon	INIDEP-T 0295	FARG296-07	EU074509	Tissue	e-voucher
Odontesthes argentinensis	Argentina, Mar Chiquita coastal lagoon	INIDEP-T 0320	FARG321-07	EU074508	Tissue	e-voucher
Odontesthes argentinensis	Argentina, Mar Chiquita coastal lagoon	INIDEP-T 0423	FARG424-08		Tissue	e-voucher
Odontesthes argentinensis	Argentina, Mar Chiquita coastal lagoon	INIDEP-T 0424	FARG425-08		Tissue	e-voucher
Odontesthes argentinensis	Argentina, Mar Chiquita coastal lagoon	INIDEP-T 0686	FARG632-09		Tissue	e-voucher
Odontesthes platensis	Argentina, off Buenos Aires province	INIDEP-T 0314	FARG315-07	EU074513	Tissue	Specimen
Oligosarcus jenynsii	Argentina, Mar Chiquita coastal lagoon	INIDEP-T 0528	FARG529-08		Tissue	e-voucher
Oligosarcus jenynsii	Argentina, Mar Chiquita coastal lagoon	INIDEP-T 0529	FARG530-08		Tissue	e-voucher
Oligosarcus jenynsii	Argentina, Mar Chiquita coastal lagoon	INIDEP-T 0530	FARG531-08		Tissue	e-voucher
Oligosarcus jenynsii	Argentina, Mar Chiquita coastal lagoon	INIDEP-T 0531	FARG532-08		Tissue	e-voucher
Oligosarcus jenynsii	Argentina, Mar Chiquita coastal lagoon	INIDEP-T 0532	FARG533-08		Tissue	e-voucher
Oncopterus darwinii	Argentina, Mar Chiquita coastal lagoon	INIDEP-T 0514	FARG515-08		Tissue	Specimen
Pagrus pagrus	Argentina, off Buenos Aires province	INIDEP-T 0483	FARG484-08		Tissue	e-voucher
Pagrus pagrus	Argentina, off Buenos Aires province	INIDEP-T 0484	FARG485-08		Tissue	e-voucher
Pagrus pagrus	Argentina, off Buenos Aires province	INIDEP-T 0491	FARG492-08		Tissue	e-voucher
Pagrus pagrus	Argentina, coastal Buenos Aires waters	INIDEP-T 0639	FARG585-09		Tissue	e-voucher
Pagrus pagrus	Argentina, coastal Buenos Aires waters	INIDEP-T 0640	FARG586-09		Tissue	e-voucher
Paralichthys isosceles	Argentina, off Patagonian waters	INIDEP-T 0058	FARG058-06	EU074514	Tissue	e-voucher
Paralichthys isosceles	Argentina, off Patagonian waters	INIDEP-T 0059	FARG059-06	EU074515	Tissue	e-voucher
Paralichthys isosceles	Argentina, off Patagonian waters	INIDEP-T 0060	FARG060-06	EU074516	Tissue	e-voucher
Paralichthys isosceles	Argentina, off Patagonian waters	INIDEP-T 0252	FARG252-06	EU074518	Tissue	e-voucher
Paralichthys isosceles	Argentina, off Patagonian waters	INIDEP-T 0253	FARG253-06	EU074517	Tissue	e-voucher
Paralichthys orbignyanus	Argentina, Mar Chiquita coastal lagoon	INIDEP-T 0285	FARG286-07	EU074520	Tissue	e-voucher
Paralichthys orbignyanus	Argentina, Mar Chiquita coastal lagoon	INIDEP-T 0321	FARG322-07	EU074519	Tissue	e-voucher
Paralichthys orbignyanus	Argentina, Mar Chiquita coastal lagoon	INIDEP-T 0421	FARG422-08		Tissue	e-voucher
Paralichthys orbignyanus	Argentina, Mar Chiquita coastal lagoon	INIDEP-T 0524	FARG525-08		Tissue	e-voucher

Paralichthys orbignyanus	Argentina, Mar Chiquita coastal lagoon	INIDEP-T 0525	FARG526-08		Tissue	e-voucher
Paralichthys orbignyanus	Argentina, Mar Chiquita coastal lagoon	INIDEP-T 0526	FARG527-08		Tissue	e-voucher
Paralichthys orbignyanus	Argentina, Mar Chiquita coastal lagoon	INIDEP-T 0643	FARG589-09		Tissue	e-voucher
Paralichthys orbignyanus	Argentina, Mar Chiquita coastal lagoon	INIDEP-T 0644	FARG590-09		Tissue	e-voucher
Paralichthys orbignyanus	Argentina, Mar Chiquita coastal lagoon	INIDEP-T 0659	FARG605-09		Tissue	e-voucher
Paralichthys orbignyanus	Argentina, Buenos Aires province coast	INIDEP-T 0700	FARG646-09		Tissue	e-voucher
Paralichthys orbignyanus	Argentina, Mar Chiquita coastal lagoon	INIDEP-T 0701	FARG647-09		Tissue	e-voucher
Paralichthys patagonicus	Argentina, off Buenos Aires province	INIDEP-T 0434	FARG435-08		Tissue	e-voucher
Paralichthys patagonicus	Argentina, off Buenos Aires province	INIDEP-T 0437	FARG438-08		Tissue	e-voucher
Paralichthys patagonicus	Argentina, off Buenos Aires province	INIDEP-T 0438	FARG439-08		Tissue	e-voucher
Paralonchurus brasiliensis	Argentina, off Buenos Aires province	INIDEP-T 0661	FARG607-09		Tissue	e-voucher
Paralonchurus brasiliensis	Argentina, off Buenos Aires province	INIDEP-T 0662	FARG608-09		Tissue	e-voucher
Paralonchurus brasiliensis	Argentina, off Buenos Aires province	INIDEP-T 0663	FARG609-09		Tissue	e-voucher
Parona signata	Argentina, off Patagonian waters	INIDEP-T 0061	FARG061-06	EU074522	Tissue	e-voucher
Parona signata	Argentina, off Patagonian waters	INIDEP-T 0062	FARG062-06	EU074523	Tissue	e-voucher
Parona signata	Argentina, off Patagonian waters	INIDEP-T 0063	FARG063-06	EU074524	Tissue	e-voucher
Parona signata	Argentina, off Patagonian waters	INIDEP-T 0064	FARG064-06	EU074525	Tissue	e-voucher
Parona signata	Argentina, Mar Chiquita coastal lagoon	INIDEP-T 0328	FARG329-07	EU074521	Tissue	e-voucher
Parona signata	Argentina, Mar Chiquita coastal lagoon	INIDEP-T 0658	FARG604-09		Tissue	e-voucher
Patagonotothen ramsayi	Argentina, off Patagonian waters	INIDEP-T 0065	FARG065-06	EU074533	Tissue	e-voucher
Patagonotothen ramsayi	Argentina, off Patagonian waters	INIDEP-T 0066	FARG066-06	EU074530	Tissue	e-voucher
Patagonotothen ramsayi	Argentina, off Patagonian waters	INIDEP-T 0067	FARG067-06	EU074531	Tissue	e-voucher
Patagonotothen ramsayi	Argentina, off Patagonian waters	INIDEP-T 0068	FARG068-06	EU074532	Tissue	e-voucher
Patagonotothen ramsayi	Argentina, off Buenos Aires province	INIDEP-T 0360	FARG361-07	EU074529	Tissue	e-voucher
Patagonotothen ramsayi	Argentina, off Buenos Aires province	INIDEP-T 0361	FARG362-07	EU074528	Tissue	e-voucher
Patagonotothen ramsayi	Argentina, off Buenos Aires province	INIDEP-T 0362	FARG363-07	EU074527	Tissue	e-voucher
Patagonotothen ramsayi	Argentina, off Buenos Aires province	INIDEP-T 0363	FARG364-07	EU074526	Tissue	e-voucher
Patagonotothen ramsayi	Argentina, off Buenos Aires province	INIDEP-T 0472	FARG473-08		Tissue	e-voucher

Patagonotothen tessellata	Argentina, off Patagonian waters	INIDEP-T 0135	FARG135-06	EU074536	Tissue	e-voucher
Patagonotothen tessellata	Argentina, off Patagonian waters	INIDEP-T 0136	FARG136-06	EU074537	Tissue	e-voucher
Patagonotothen tessellata	Argentina, off Patagonian waters	INIDEP-T 0137	FARG137-06	EU074538	Tissue	e-voucher
Patagonotothen tessellata	Argentina, off Patagonian waters	INIDEP-T 0138	FARG138-06	EU074534	Tissue	e-voucher
Patagonotothen tessellata	Argentina, off Patagonian waters	INIDEP-T 0152	FARG152-06	EU074535	Tissue	e-voucher
Peprilus paru	Argentina, Buenos Aires province coast	INIDEP-T 0588	FARG563-09		Tissue	e-voucher
Peprilus paru	Argentina, Buenos Aires province coast	INIDEP-T 0589	FARG564-09		Tissue	e-voucher
Percophis brasiliensis	Argentina, off Patagonian waters	INIDEP-T 0083	FARG083-06	EU074541	Tissue	e-voucher
Percophis brasiliensis	Argentina, coastal Patagonian waters	INIDEP-T 0084	FARG084-06	EU074540	Tissue	e-voucher
Percophis brasiliensis	Argentina, coastal Patagonian waters	INIDEP-T 0085	FARG085-06	EU074539	Tissue	e-voucher
Percophis brasiliensis	Argentina, off Buenos Aires province	INIDEP-T 0431	FARG432-08		Tissue	e-voucher
Percophis brasiliensis	Argentina, off Buenos Aires province	INIDEP-T 0436	FARG437-08		Tissue	e-voucher
Percophis brasiliensis	Argentina, off Buenos Aires province	INIDEP-T 0475	FARG476-08		Tissue	e-voucher
Percophis brasiliensis	Argentina, off Buenos Aires province	INIDEP-T 0502	FARG503-08		Tissue	e-voucher
Percophis brasiliensis	Argentina, Buenos Aires province coast	INIDEP-T 0699	FARG645-09		Tissue	e-voucher
Pinguipes brasilianus	Argentina, off Patagonian waters	INIDEP-T 0086	FARG086-06	EU074542	Tissue	e-voucher
Pinguipes brasilianus	Argentina, off Buenos Aires province	INIDEP-T 0464	FARG465-08		Tissue	e-voucher
Platanichthys platana	Argentina, Mar Chiquita coastal lagoon	INIDEP-T 0416	FARG417-08		Tissue	e-voucher
Platanichthys platana	Argentina, Mar Chiquita coastal lagoon	INIDEP-T 0417	FARG418-08		Tissue	e-voucher
Platanichthys platana	Argentina, Mar Chiquita coastal lagoon	INIDEP-T 0418	FARG419-08		Tissue	e-voucher
Platanichthys platana	Argentina, Mar Chiquita coastal lagoon	INIDEP-T 0419	FARG420-08		Tissue	e-voucher
Platanichthys platana	Argentina, Mar Chiquita coastal lagoon	INIDEP-T 0420	FARG421-08		Tissue	e-voucher
Platanichthys platana	Argentina, Mar Chiquita coastal lagoon	INIDEP-T 0429	FARG430-08		Tissue	e-voucher
Pogonias cromis	Argentina, Mar Chiquita coastal lagoon	INIDEP-T 0287	FARG288-07	EU074550	Tissue	e-voucher
Pogonias cromis	Argentina, Mar Chiquita coastal lagoon	INIDEP-T 0292	FARG293-07	EU074549	Tissue	e-voucher
Pogonias cromis	Argentina, Mar Chiquita coastal lagoon	INIDEP-T 0298	FARG299-07	EU074548	Tissue	e-voucher
Pogonias cromis	Argentina, Mar Chiquita coastal lagoon	INIDEP-T 0299	FARG300-07	EU074547	Tissue	e-voucher
Pogonias cromis	Argentina, Mar Chiquita coastal lagoon	INIDEP-T 0300	FARG301-07	EU074546	Tissue	e-voucher

Pogonias cromis	Argentina, Mar Chiquita coastal lagoon	INIDEP-T 0301	FARG302-07	EU074545	Tissue	e-voucher
Pogonias cromis	Argentina, Mar Chiquita coastal lagoon	INIDEP-T 0302	FARG303-07	EU074544	Tissue	e-voucher
Pogonias cromis	Argentina, Mar Chiquita coastal lagoon	INIDEP-T 0303	FARG304-07	EU074543	Tissue	e-voucher
Pogonias cromis	Argentina, Mar Chiquita coastal lagoon	INIDEP-T 0650	FARG596-09		Tissue	e-voucher
Pogonias cromis	Argentina, Mar Chiquita coastal lagoon	INIDEP-T 0651	FARG597-09		Tissue	e-voucher
Pogonias cromis	Argentina, Mar Chiquita coastal lagoon	INIDEP-T 0652	FARG598-09		Tissue	e-voucher
Polyprion americanus	Argentina, off Buenos Aires province	INIDEP-T 0674	FARG620-09		Tissue	e-voucher
Polyprion americanus	Argentina, off Buenos Aires province	INIDEP-T 0675	FARG621-09		Tissue	e-voucher
Polyprion americanus	Argentina, off Buenos Aires province	INIDEP-T 0676	FARG622-09		Tissue	e-voucher
Pomatomus saltatrix	Argentina, Mar Chiquita coastal lagoon	INIDEP-T 0296	FARG297-07	EU074555	Tissue	e-voucher
Pomatomus saltatrix	Argentina, Mar Chiquita coastal lagoon	INIDEP-T 0308	FARG309-07	EU074554	Tissue	e-voucher
Pomatomus saltatrix	Argentina, Mar Chiquita coastal lagoon	INIDEP-T 0309	FARG310-07	EU074553	Tissue	e-voucher
Pomatomus saltatrix	Argentina, Mar Chiquita coastal lagoon	INIDEP-T 0310	FARG311-07	EU074552	Tissue	e-voucher
Pomatomus saltatrix	Argentina, Mar Chiquita coastal lagoon	INIDEP-T 0311	FARG312-07	EU074551	Tissue	e-voucher
Porichthys porosissimus	Argentina, Buenos Aires province coast	INIDEP-T 0551	FARG552-08		Tissue	e-voucher
Prionotus nudigula	Argentina, off Buenos Aires province	INIDEP-T 0433	FARG434-08		Tissue	e-voucher
Prionotus nudigula	Argentina, off Buenos Aires province	INIDEP-T 0477	FARG478-08		Tissue	e-voucher
Prionotus nudigula	Argentina, off Buenos Aires province	INIDEP-T 0490	FARG491-08		Tissue	e-voucher
Prionotus nudigula	Argentina, off Buenos Aires province	INIDEP-T 0495	FARG496-08		Tissue	e-voucher
Prionotus punctatus	Argentina, Buenos Aires province coast	INIDEP-T 0634	FARG580-09		Tissue	e-voucher
Prionotus punctatus	Argentina, Buenos Aires province coast	INIDEP-T 0635	FARG581-09		Tissue	e-voucher
Psammobatis lentiginosa	Argentina, off Buenos Aires province	INIDEP-T 0347	FARG348-07	EU074559	Tissue	e-voucher
Psammobatis lentiginosa	Argentina, off Buenos Aires province	INIDEP-T 0348	FARG349-07	EU074558	Tissue	e-voucher
Psammobatis lentiginosa	Argentina, off Buenos Aires province	INIDEP-T 0349	FARG350-07	EU074557	Tissue	e-voucher
Psammobatis lentiginosa	Argentina, off Buenos Aires province	INIDEP-T 0350	FARG351-07	EU074556	Tissue	e-voucher
Psammobatis lentiginosa	Argentina, off Buenos Aires province	INIDEP-T 0462	FARG463-08		Tissue	e-voucher
Psammobatis lentiginosa	Argentina, off Buenos Aires province	INIDEP-T 0463	FARG464-08		Tissue	e-voucher
Psammobatis normani	Argentina, off Patagonian waters	INIDEP-T 0376	FARG377-08		Tissue	e-voucher

Psammobatis normani	Argentina, off Buenos Aires province	INIDEP-T 0470	FARG471-08		Tissue	e-voucher
Psammobatis normani	Argentina, off Buenos Aires province	INIDEP-T 0473	FARG474-08		Tissue	e-voucher
Psammobatis rudis	Argentina, off Patagonian waters	INIDEP-T 0070	FARG070-06	EU074569	Tissue	e-voucher
Psammobatis rudis	Argentina, off Patagonian waters	INIDEP-T 0071	FARG071-06	EU074566	Tissue	e-voucher
Psammobatis rudis	Argentina, off Patagonian waters	INIDEP-T 0072	FARG072-06	EU074563	Tissue	e-voucher
Psammobatis rudis	Argentina, off Patagonian waters	INIDEP-T 0073	FARG073-06	EU074562	Tissue	e-voucher
Psammobatis rudis	Argentina, off Patagonian waters	INIDEP-T 0074	FARG074-06	EU074565	Tissue	e-voucher
Psammobatis rudis	Argentina, off Patagonian waters	INIDEP-T 0075	FARG075-06	EU074567	Tissue	e-voucher
Psammobatis rudis	Argentina, off Patagonian waters	INIDEP-T 0076	FARG076-06	EU074568	Tissue	e-voucher
Psammobatis rudis	Argentina, off Patagonian waters	INIDEP-T 0077	FARG077-06	EU074564	Tissue	e-voucher
Psammobatis rudis	Argentina, off Patagonian waters	INIDEP-T 0122	FARG122-06	EU074560	Tissue	e-voucher
Psammobatis rudis	Argentina, off Patagonian waters	INIDEP-T 0123	FARG123-06	EU074561	Tissue	e-voucher
Psammobatis rudis	Argentina, off Patagonian waters	INIDEP-T 0377	FARG378-08		Tissue	e-voucher
Psammobatis rudis	Argentina, off Patagonian waters	INIDEP-T 0378	FARG379-08		Tissue	e-voucher
Psammobatis rudis	Argentina, off Patagonian waters	INIDEP-T 0389	FARG390-08		Tissue	e-voucher
Psammobatis rudis	Argentina, off Patagonian waters	INIDEP-T 0390	FARG391-08		Tissue	e-voucher
Psammobatis rudis	Argentina, off Patagonian waters	INIDEP-T 0391	FARG392-08		Tissue	e-voucher
Psammobatis rudis	Argentina, off Buenos Aires province	INIDEP-T 0468	FARG469-08		Tissue	e-voucher
Psammobatis rudis	Argentina, off Buenos Aires province	INIDEP-T 0478	FARG479-08		Tissue	e-voucher
Psammobatis rudis	Argentina, off Buenos Aires province	INIDEP-T 0493	FARG494-08		Tissue	e-voucher
Pseudocyttus maculatus	Argentina, off Patagonian waters	INIDEP-T 0276	FARG277-07	EU074570	Tissue	e-voucher
Pseudopercis semifasciata	Argentina, off Patagonian waters	INIDEP-T 0078	FARG078-06	EU074574	Tissue	e-voucher
Pseudopercis semifasciata	Argentina, off Patagonian waters	INIDEP-T 0079	FARG079-06	EU074571	Tissue	e-voucher
Pseudopercis semifasciata	Argentina, off Patagonian waters	INIDEP-T 0080	FARG080-06	EU074572	Tissue	e-voucher
Pseudopercis semifasciata	Argentina, off Patagonian waters	INIDEP-T 0081	FARG081-06	EU074573	Tissue	e-voucher
Pseudopercis semifasciata	Argentina, off Patagonian waters	INIDEP-T 0082	FARG082-06	EU074575	Tissue	e-voucher
Pseudopercis semifasciata	Argentina, off Buenos Aires province	INIDEP-T 0482	FARG483-08		Tissue	e-voucher
Psychrolutes marmoratus	Argentina, off Patagonian waters	INIDEP-T 0139	FARG139-06	EU074576	Tissue	e-voucher

Raneya brasiliensis	Argentina, off Patagonian waters	INIDEP-T 0087	FARG087-06	EU074577	Tissue	e-voucher
Raneya brasiliensis	Argentina, off Patagonian waters	INIDEP-T 0088	FARG088-06	EU074578	Tissue	e-voucher
Rhamdia sapo	Argentina, Mar Chiquita coastal lagoon	INIDEP-T 0316	FARG317-07	EU074579	Tissue	e-voucher
Rioraja agassizii	Argentina, off Buenos Aires province	INIDEP-T 0404	FARG405-08		Tissue	e-voucher
Rioraja agassizii	Argentina, off Buenos Aires province	INIDEP-T 0408	FARG409-08		Tissue	e-voucher
Salilota australis	Argentina, off Patagonian waters	INIDEP-T 0090	FARG090-06	EU074580	Tissue	e-voucher
Salilota australis	Argentina, off Patagonian waters	INIDEP-T 0374	FARG375-08		Tissue	e-voucher
Schroederichthys bivius	Argentina, off Patagonian waters	INIDEP-T 0095	FARG095-06	EU074586	Tissue	e-voucher
Schroederichthys bivius	Argentina, off Patagonian waters	INIDEP-T 0096	FARG096-06	EU074581	Tissue	e-voucher
Schroederichthys bivius	Argentina, off Patagonian waters	INIDEP-T 0189	FARG189-06	EU074585	Tissue	e-voucher
Schroederichthys bivius	Argentina, off Patagonian waters	INIDEP-T 0190	FARG190-06	EU074584	Tissue	e-voucher
Schroederichthys bivius	Uruguay, common fishing area Argentina-Uruguay	INIDEP-T 0335	FARG336-07	EU074583	Tissue	e-voucher
Schroederichthys bivius	Uruguay, common fishing area Argentina-Uruguay	INIDEP-T 0336	FARG337-07	EU074582	Tissue	e-voucher
Schroederichthys bivius	Argentina, off Patagonian waters	INIDEP-T 0365	FARG366-08		Tissue	e-voucher
Schroederichthys bivius	Argentina, off Patagonian waters	INIDEP-T 0366	FARG367-08		Tissue	e-voucher
Scomber japonicus	Argentina, off Buenos Aires province	INIDEP-T 0480	FARG481-08		Tissue	e-voucher
Scomber japonicus	Argentina, off Buenos Aires province	INIDEP-T 0481	FARG482-08		Tissue	e-voucher
Scomber japonicus	Argentina, off Buenos Aires province	INIDEP-T 0485	FARG486-08		Tissue	e-voucher
Scomber japonicus	Argentina, off Buenos Aires province	INIDEP-T 0486	FARG487-08		Tissue	e-voucher
Scomber japonicus	Argentina, off Buenos Aires province	INIDEP-T 0494	FARG495-08		Tissue	e-voucher
Sebastes oculatus	Argentina, off Patagonian waters	INIDEP-T 0191	FARG191-06	EU074589	Tissue	e-voucher
Sebastes oculatus	Argentina, off Patagonian waters	INIDEP-T 0192	FARG192-06	EU074590	Tissue	e-voucher
Sebastes oculatus	Argentina, off Patagonian waters	INIDEP-T 0193	FARG193-06	EU074587	Tissue	e-voucher
Sebastes oculatus	Argentina, off Patagonian waters	INIDEP-T 0194	FARG194-06	EU074588	Tissue	e-voucher
Sebastes oculatus	Argentina, off Patagonian waters	INIDEP-T 0195	FARG195-06	EU074591	Tissue	e-voucher
Selene setapinnis	Argentina, Buenos Aires province coast	INIDEP-T 0587	FARG562-09		Tissue	Specimen

Selene vomer	Argentina, Mar Chiquita coastal lagoon	INIDEP-T 0523	FARG524-08		Tissue	Specimen
Seriolella caerulea	Argentina, off Patagonian waters	INIDEP-T 0126	FARG126-06	EU074592	Tissue	e-voucher
Seriolella caerulea	Argentina, off Patagonian waters	INIDEP-T 0401	FARG402-08		Tissue	e-voucher
Seriolella porosa	Argentina, off Patagonian waters	INIDEP-T 0196	FARG196-06	EU074595	Tissue	e-voucher
Seriolella porosa	Argentina, off Patagonian waters	INIDEP-T 0197	FARG197-06	EU074594	Tissue	e-voucher
Seriolella porosa	Argentina, off Patagonian waters	INIDEP-T 0198	FARG198-06	EU074593	Tissue	e-voucher
Seriolella porosa	Argentina, off Buenos Aires province	INIDEP-T 0402	FARG403-08		Tissue	e-voucher
Serranus auriga	Argentina, Buenos Aires province coast	INIDEP-T 0591	FARG566-09		Tissue	e-voucher
Serranus auriga	Argentina, Buenos Aires province coast	INIDEP-T 0696	FARG642-09		Tissue	e-voucher
Sphoeroides pachygaster	Uruguay, common fishing area Argentina-Uruguay	INIDEP-T 0339	FARG340-07	EU074598	Tissue	e-voucher
Sphoeroides pachygaster	Uruguay, common fishing area Argentina-Uruguay	INIDEP-T 0340	FARG341-07	EU074597	Tissue	e-voucher
Sphoeroides pachygaster	Uruguay, common fishing area Argentina-Uruguay	INIDEP-T 0341	FARG342-07	EU074596	Tissue	e-voucher
Sphyraena guachancho	Argentina, coastal Buenos Aires province	INIDEP-T 0542	FARG543-08		Tissue	e-voucher
Squalus acanthias	Argentina, off Patagonian waters	INIDEP-T 0199	FARG199-06	EU074609	Tissue	e-voucher
Squalus acanthias	Argentina, off Patagonian waters	INIDEP-T 0201	FARG201-06	EU074607	Tissue	e-voucher
Squalus acanthias	Argentina, off Patagonian waters	INIDEP-T 0202	FARG202-06	EU074601	Tissue	e-voucher
Squalus acanthias	Argentina, off Patagonian waters	INIDEP-T 0203	FARG203-06	EU074605	Tissue	e-voucher
Squalus acanthias	Argentina, off Patagonian waters	INIDEP-T 0204	FARG204-06	EU074606	Tissue	e-voucher
Squalus acanthias	Argentina, off Patagonian waters	INIDEP-T 0205	FARG205-06	EU074608	Tissue	e-voucher
Squalus acanthias	Argentina, off Patagonian waters	INIDEP-T 0227	FARG227-06	EU074603	Tissue	e-voucher
Squalus acanthias	Argentina, off Patagonian waters	INIDEP-T 0228	FARG228-06	EU074604	Tissue	e-voucher
Squalus acanthias	Argentina, off Patagonian waters	INIDEP-T 0254	FARG254-06	EU074600	Tissue	e-voucher
Squalus acanthias	Argentina, off Patagonian waters	INIDEP-T 0255	FARG255-06	EU074599	Tissue	e-voucher

Squalus acanthias	Uruguay, common fishing area Argentina-Uruguay	INIDEP-T 0333	FARG334-07	EU074602	Tissue	e-voucher
Squalus mitsukurii	Uruguay, common fishing area Argentina-Uruguay	INIDEP-T 0332	FARG333-07	EU074611	Tissue	e-voucher
Squalus mitsukurii	Uruguay, common fishing area Argentina-Uruguay	INIDEP-T 0334	FARG335-07	EU074610	Tissue	e-voucher
Squatina guggenheim	Argentina, off Buenos Aires province	INIDEP-T 0467	FARG468-08		Tissue	e-voucher
Squatina guggenheim	Argentina, off Buenos Aires province	INIDEP-T 0488	FARG489-08		Tissue	e-voucher
Squatina guggenheim	Argentina, off Buenos Aires province	INIDEP-T 0497	FARG498-08		Tissue	e-voucher
Squatina guggenheim	Argentina, off Buenos Aires province	INIDEP-T 0656	FARG602-09		Tissue	e-voucher
Squatina guggenheim	Argentina, off Buenos Aires province	INIDEP-T 0657	FARG603-09		Tissue	e-voucher
Stromateus brasiliensis	Argentina, off Patagonian waters	INIDEP-T 0207	FARG207-06	EU074612	Tissue	e-voucher
Stromateus brasiliensis	Argentina, off Patagonian waters	INIDEP-T 0208	FARG208-06	EU074613	Tissue	e-voucher
Stromateus brasiliensis	Argentina, off Patagonian waters	INIDEP-T 0209	FARG209-06	EU074614	Tissue	e-voucher
Stromateus brasiliensis	Argentina, off Patagonian waters	INIDEP-T 0210	FARG210-06	EU074615	Tissue	e-voucher
Stromateus brasiliensis	Argentina, off Patagonian waters	INIDEP-T 0392	FARG393-08		Tissue	e-voucher
Stromateus brasiliensis	Argentina, off Patagonian waters	INIDEP-T 0393	FARG394-08		Tissue	e-voucher
Stromateus brasiliensis	Argentina, off Patagonian waters	INIDEP-T 0394	FARG395-08		Tissue	e-voucher
Sympterygia acuta	Argentina, off Buenos Aires province	INIDEP-T 0405	FARG406-08		Tissue	e-voucher
Sympterygia acuta	Argentina, off Buenos Aires province	INIDEP-T 0407	FARG408-08		Tissue	e-voucher
Sympterygia bonapartii	Argentina, off Patagonian waters	INIDEP-T 0211	FARG211-06	EU074617	Tissue	e-voucher
Sympterygia bonapartii	Argentina, off Patagonian waters	INIDEP-T 0212	FARG212-06	EU074620	Tissue	e-voucher
Sympterygia bonapartii	Argentina, off Patagonian waters	INIDEP-T 0213	FARG213-06	EU074618	Tissue	e-voucher
Sympterygia bonapartii	Argentina, off Patagonian waters	INIDEP-T 0214	FARG214-06	EU074616	Tissue	e-voucher
Sympterygia bonapartii	Argentina, off Patagonian waters	INIDEP-T 0215	FARG215-06	EU074619	Tissue	e-voucher
Trachurus lathami	Argentina, off Buenos Aires province	INIDEP-T 0459	FARG460-08		Tissue	e-voucher
Trachurus lathami	Argentina, Mar Chiquita coastal lagoon	INIDEP-T 0552	FARG553-08		Tissue	e-voucher
Triathalassothia argentina	Argentina, Buenos Aires province coast	INIDEP-T 0549	FARG550-08		Tissue	e-voucher

Triathalassothia argentina	Argentina, Buenos Aires province coast	INIDEP-T 0550	FARG551-08		Tissue	e-voucher
Triathalassothia argentina	Argentina, Buenos Aires province coast	INIDEP-T 0636	FARG582-09		Tissue	e-voucher
Urophycis brasiliensis	Argentina, Mar Chiquita coastal lagoon	INIDEP-T 0653	FARG599-09		Tissue	e-voucher
Urophycis cirrata	Argentina, off La Plata River	INIDEP-T 0344	FARG345-07	EU074621	Tissue	e-voucher
Xystreurys rasile	Argentina, off Patagonian waters	INIDEP-T 0216	FARG216-06	EU074625	Tissue	e-voucher
Xystreurys rasile	Argentina, off Patagonian waters	INIDEP-T 0217	FARG217-06	EU074624	Tissue	e-voucher
Xystreurys rasile	Argentina, off Patagonian waters	INIDEP-T 0218	FARG218-06	EU074629	Tissue	e-voucher
Xystreurys rasile	Argentina, off Patagonian waters	INIDEP-T 0219	FARG219-06	EU074626	Tissue	e-voucher
Xystreurys rasile	Argentina, off Patagonian waters	INIDEP-T 0220	FARG220-06	EU074627	Tissue	e-voucher
Xystreurys rasile	Argentina, off Patagonian waters	INIDEP-T 0221	FARG221-06	EU074628	Tissue	e-voucher
Xystreurys rasile	Argentina, off Buenos Aires province	INIDEP-T 0357	FARG358-07	EU074623	Tissue	e-voucher
Xystreurys rasile	Argentina, off Buenos Aires province	INIDEP-T 0358	FARG359-07	EU074622	Tissue	e-voucher
Zenopsis conchifera	Argentina, Buenos Aires province coast	INIDEP-T 0590	FARG565-09		Tissue	e-voucher
