# Supplementary material for: DNA Barcoding Identifies Argentine Fishes from Marine and Brackish Waters
Source: PLoS One. 2011 Dec 9;6(12):e28655. doi: 10.1371/journal.pone.0028655 (PMC3235135; doi:10.1371/journal.pone.0028655)
Supplement: Appendix S1 — Neighbour-joining tree of 577 COI sequences from the 125 fish species sampled as obtained in BOLD, using K2P distances. (PDF) [file pone.0028655.s003.pdf]

BOLD TaxonID Tree

Project	:	Fishes of Argentina [FARG]
Date	:	4-March-2010
Data Type	:	Nucleotide
Distance Model	:	Kimura 2 Parameter
Codon Positions	:	1st, 2nd, 3rd
Labels	:	ProcessID, Family,
Colorization	:	

Sequence Count	:	577
Species count	:	126
Genus count	:	98
Family count	:	63
Unidentified	:	0

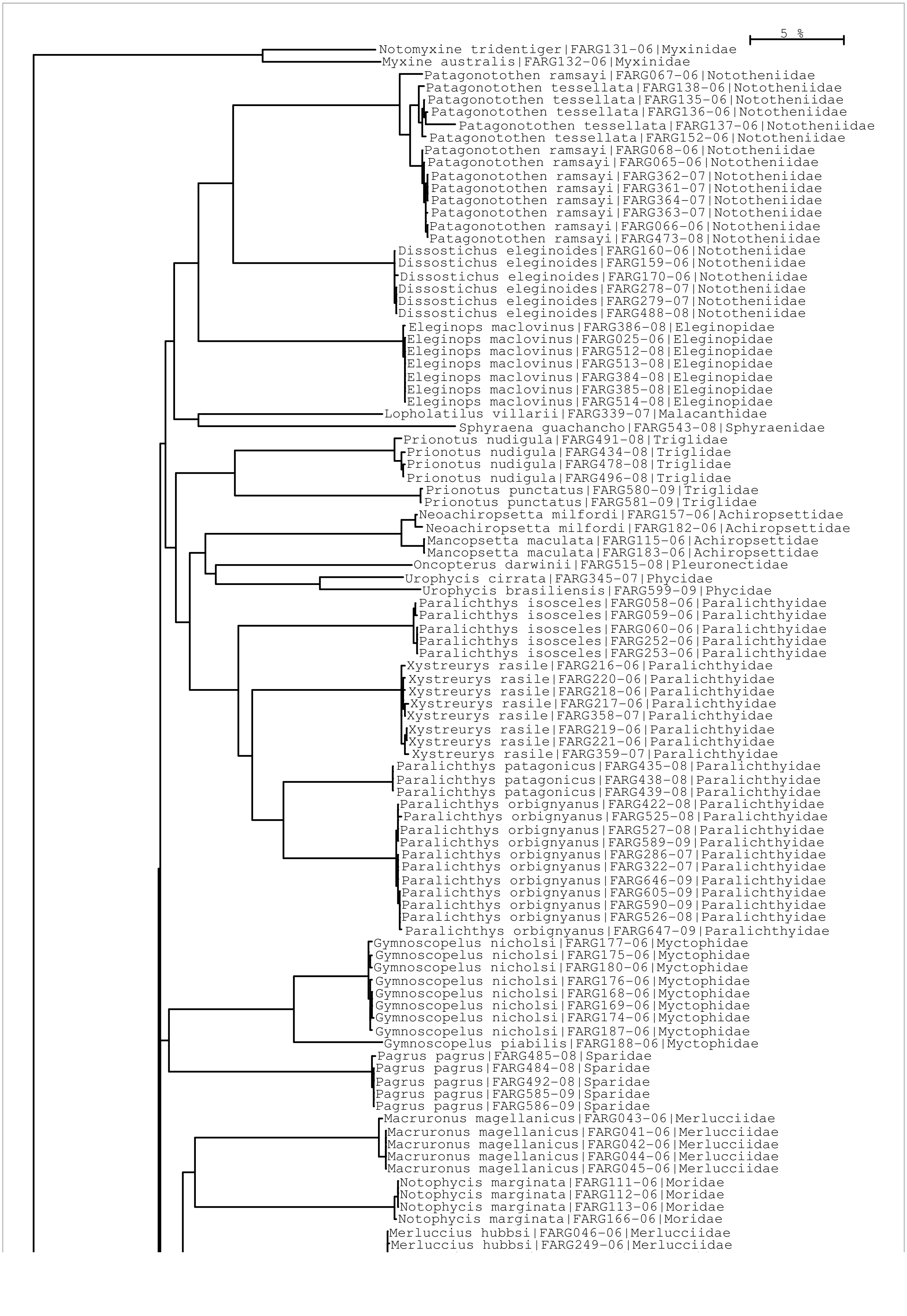
5 %
Notomyxine tridentiger|FARG131-06|Myxinidae
Myxine australis|FARG132-06|Myxinidae
Patagonotothen ramsayi|FARG067-06|Nototheniidae
Patagonotothen tessellata|FARG138-06|Nototheniidae
Patagonotothen tessellata|FARG135-06|Nototheniidae
Patagonotothen tessellata|FARG136-06|Nototheniidae
Patagonotothen tessellata|FARG137-06|Nototheniidae
Patagonotothen tessellata|FARG152-06|Nototheniidae
Patagonotothen ramsayi|FARG068-06|Nototheniidae
Patagonotothen ramsayi|FARG065-06|Nototheniidae
Patagonotothen ramsayi|FARG362-07|Nototheniidae
Patagonotothen ramsayi|FARG361-07|Nototheniidae
Patagonotothen ramsayi|FARG364-07|Nototheniidae
Patagonotothen ramsayi|FARG363-07|Nototheniidae
Patagonotothen ramsayi|FARG066-06|Nototheniidae
Patagonotothen ramsayi|FARG473-08|Nototheniidae
Dissostichus eleginoides|FARG160-06|Nototheniidae
Dissostichus eleginoides|FARG159-06|Nototheniidae
Dissostichus eleginoides|FARG170-06|Nototheniidae
Dissostichus eleginoides|FARG278-07|Nototheniidae
Dissostichus eleginoides|FARG279-07|Nototheniidae
Dissostichus eleginoides|FARG488-08|Nototheniidae
Eleginops maclovinus|FARG386-08|Eleginopidae
Eleginops maclovinus|FARG025-06|Eleginopidae
Eleginops maclovinus|FARG512-08|Eleginopidae
Eleginops maclovinus|FARG513-08|Eleginopidae
Eleginops maclovinus|FARG384-08|Eleginopidae
Eleginops maclovinus|FARG385-08|Eleginopidae
Eleginops maclovinus|FARG514-08|Eleginopidae
Lopholatilus villarii|FARG339-07|Malacanthidae
Sphyraena guachancho|FARG543-08|Sphyraenidae
Prionotus nudigula|FARG491-08|Triglidae
Prionotus nudigula|FARG434-08|Triglidae
Prionotus nudigula|FARG478-08|Triglidae
Prionotus nudigula|FARG496-08|Triglidae
Prionotus punctatus|FARG580-09|Triglidae
Prionotus punctatus|FARG581-09|Triglidae
Neoachiropsetta milfordi|FARG157-06|Achiropsettidae
Neoachiropsetta milfordi|FARG182-06|Achiropsettidae
Mancopsetta maculata|FARG115-06|Achiropsettidae
Mancopsetta maculata|FARG183-06|Achiropsettidae
Oncopterus darwinii|FARG515-08|Pleuronectidae
Urophycis cirrata|FARG345-07|Phycidae
Urophycis brasiliensis|FARG599-09|Phycidae
Paralichthys isosceles|FARG058-06|Paralichthyidae
Paralichthys isosceles|FARG059-06|Paralichthyidae
Paralichthys isosceles|FARG060-06|Paralichthyidae
Paralichthys isosceles|FARG252-06|Paralichthyidae
Paralichthys isosceles|FARG253-06|Paralichthyidae
Xystreurys rasile|FARG216-06|Paralichthyidae
Xystreurys rasile|FARG220-06|Paralichthyidae
Xystreurys rasile|FARG218-06|Paralichthyidae
Xystreurys rasile|FARG217-06|Paralichthyidae
Xystreurys rasile|FARG358-07|Paralichthyidae
Xystreurys rasile|FARG219-06|Paralichthyidae
Xystreurys rasile|FARG221-06|Paralichthyidae
Xystreurys rasile|FARG359-07|Paralichthyidae
Paralichthys patagonicus|FARG435-08|Paralichthyidae
Paralichthys patagonicus|FARG438-08|Paralichthyidae
Paralichthys patagonicus|FARG439-08|Paralichthyidae
Paralichthys orbignyanus|FARG422-08|Paralichthyidae
Paralichthys orbignyanus|FARG525-08|Paralichthyidae
Paralichthys orbignyanus|FARG527-08|Paralichthyidae
Paralichthys orbignyanus|FARG589-09|Paralichthyidae
Paralichthys orbignyanus|FARG286-07|Paralichthyidae
Paralichthys orbignyanus|FARG322-07|Paralichthyidae
Paralichthys orbignyanus|FARG646-09|Paralichthyidae
Paralichthys orbignyanus|FARG605-09|Paralichthyidae
Paralichthys orbignyanus|FARG590-09|Paralichthyidae
Paralichthys orbignyanus|FARG526-08|Paralichthyidae
Paralichthys orbignyanus|FARG647-09|Paralichthyidae
Gymnoscopelus nicholsi|FARG177-06|Myctophidae
Gymnoscopelus nicholsi|FARG175-06|Myctophidae
Gymnoscopelus nicholsi|FARG180-06|Myctophidae
Gymnoscopelus nicholsi|FARG176-06|Myctophidae
Gymnoscopelus nicholsi|FARG168-06|Myctophidae
Gymnoscopelus nicholsi|FARG169-06|Myctophidae
Gymnoscopelus nicholsi|FARG174-06|Myctophidae
Gymnoscopelus nicholsi|FARG187-06|Myctophidae
Gymnoscopelus piabilis|FARG188-06|Myctophidae
Pagrus pagrus|FARG485-08|Sparidae
Pagrus pagrus|FARG484-08|Sparidae
Pagrus pagrus|FARG492-08|Sparidae
Pagrus pagrus|FARG585-09|Sparidae
Pagrus pagrus|FARG586-09|Sparidae
Macruronus magellanicus|FARG043-06|Merlucciidae
Macruronus magellanicus|FARG041-06|Merlucciidae
Macruronus magellanicus|FARG042-06|Merlucciidae
Macruronus magellanicus|FARG044-06|Merlucciidae
Macruronus magellanicus|FARG045-06|Merlucciidae
Notophycis marginata|FARG111-06|Moridae
Notophycis marginata|FARG112-06|Moridae
Notophycis marginata|FARG113-06|Moridae
Notophycis marginata|FARG166-06|Moridae
Merluccius hubbsi|FARG046-06|Merlucciidae
Merluccius hubbsi|FARG249-06|Merlucciidae

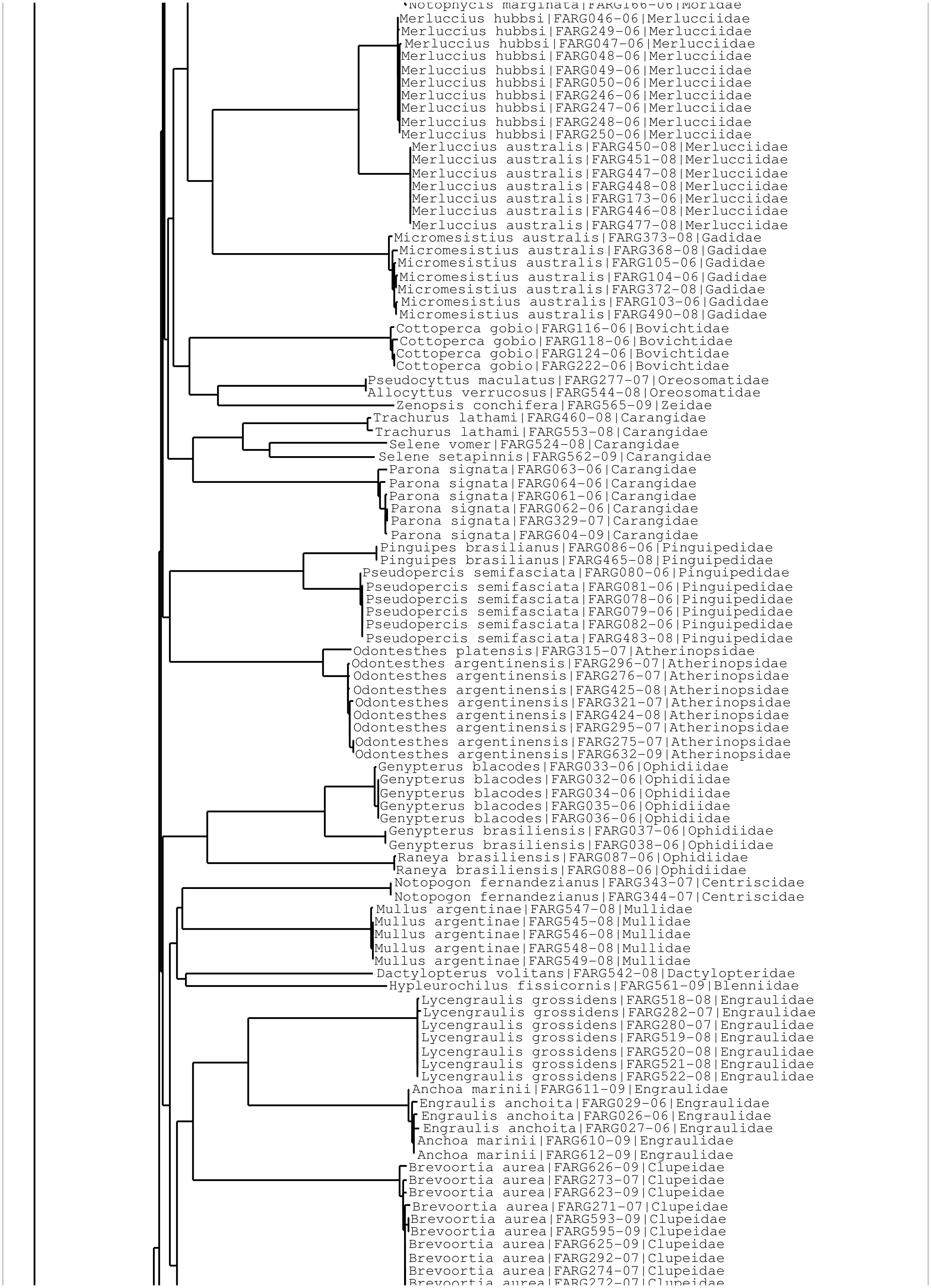
Notophycis marginata|FARG166-06|Moridae
Merluccius hubbsi|FARG046-06|Merlucciidae
Merluccius hubbsi|FARG249-06|Merlucciidae
Merluccius hubbsi|FARG047-06|Merlucciidae
Merluccius hubbsi|FARG048-06|Merlucciidae
Merluccius hubbsi|FARG049-06|Merlucciidae
Merluccius hubbsi|FARG050-06|Merlucciidae
Merluccius hubbsi|FARG246-06|Merlucciidae
Merluccius hubbsi|FARG247-06|Merlucciidae
Merluccius hubbsi|FARG248-06|Merlucciidae
Merluccius hubbsi|FARG250-06|Merlucciidae
Merluccius australis|FARG450-08|Merlucciidae
Merluccius australis|FARG451-08|Merlucciidae
Merluccius australis|FARG447-08|Merlucciidae
Merluccius australis|FARG448-08|Merlucciidae
Merluccius australis|FARG173-06|Merlucciidae
Merluccius australis|FARG446-08|Merlucciidae
Merluccius australis|FARG477-08|Merlucciidae
Micromesistius australis|FARG373-08|Gadidae
Micromesistius australis|FARG368-08|Gadidae
Micromesistius australis|FARG105-06|Gadidae
Micromesistius australis|FARG104-06|Gadidae
Micromesistius australis|FARG372-08|Gadidae
Micromesistius australis|FARG103-06|Gadidae
Micromesistius australis|FARG490-08|Gadidae
Cottoperca gobio|FARG116-06|Bovichtidae
Cottoperca gobio|FARG118-06|Bovichtidae
Cottoperca gobio|FARG124-06|Bovichtidae
Cottoperca gobio|FARG222-06|Bovichtidae
Pseudocyttus maculatus|FARG277-07|Oreosomatidae
Allocyttus verrucosus|FARG544-08|Oreosomatidae
Zenopsis conchifera|FARG565-09|Zeidae
Trachurus lathami|FARG460-08|Carangidae
Trachurus lathami|FARG553-08|Carangidae
Selene vomer|FARG524-08|Carangidae
Selene setapinnis|FARG562-09|Carangidae
Parona signata|FARG063-06|Carangidae
Parona signata|FARG064-06|Carangidae
Parona signata|FARG061-06|Carangidae
Parona signata|FARG062-06|Carangidae
Parona signata|FARG329-07|Carangidae
Parona signata|FARG604-09|Carangidae
Pinguipes brasilianus|FARG086-06|Pinguipedidae
Pinguipes brasilianus|FARG465-08|Pinguipedidae
Pseudopercis semifasciata|FARG080-06|Pinguipedidae
Pseudopercis semifasciata|FARG081-06|Pinguipedidae
Pseudopercis semifasciata|FARG078-06|Pinguipedidae
Pseudopercis semifasciata|FARG079-06|Pinguipedidae
Pseudopercis semifasciata|FARG082-06|Pinguipedidae
Pseudopercis semifasciata|FARG483-08|Pinguipedidae
Odontesthes platensis|FARG315-07|Atherinopsidae
Odontesthes argentinensis|FARG296-07|Atherinopsidae
Odontesthes argentinensis|FARG276-07|Atherinopsidae
Odontesthes argentinensis|FARG425-08|Atherinopsidae
Odontesthes argentinensis|FARG321-07|Atherinopsidae
Odontesthes argentinensis|FARG424-08|Atherinopsidae
Odontesthes argentinensis|FARG295-07|Atherinopsidae
Odontesthes argentinensis|FARG275-07|Atherinopsidae
Odontesthes argentinensis|FARG632-09|Atherinopsidae
Genypterus blacodes|FARG033-06|Ophidiidae
Genypterus blacodes|FARG032-06|Ophidiidae
Genypterus blacodes|FARG034-06|Ophidiidae
Genypterus blacodes|FARG035-06|Ophidiidae
Genypterus blacodes|FARG036-06|Ophidiidae
Genypterus brasiliensis|FARG037-06|Ophidiidae
Genypterus brasiliensis|FARG038-06|Ophidiidae
Raneya brasiliensis|FARG087-06|Ophidiidae
Raneya brasiliensis|FARG088-06|Ophidiidae
Notopogon fernandezianus|FARG343-07|Centriscidae
Notopogon fernandezianus|FARG344-07|Centriscidae
Mullus argentinae|FARG547-08|Mullidae
Mullus argentinae|FARG545-08|Mullidae
Mullus argentinae|FARG546-08|Mullidae
Mullus argentinae|FARG548-08|Mullidae
Mullus argentinae|FARG549-08|Mullidae
Dactylopterus volitans|FARG542-08|Dactylopteridae
Hypleurochilus fissicornis|FARG561-09|Blenniidae
Lycengraulis grossidens|FARG518-08|Engraulidae
Lycengraulis grossidens|FARG282-07|Engraulidae
Lycengraulis grossidens|FARG280-07|Engraulidae
Lycengraulis grossidens|FARG519-08|Engraulidae
Lycengraulis grossidens|FARG520-08|Engraulidae
Lycengraulis grossidens|FARG521-08|Engraulidae
Lycengraulis grossidens|FARG522-08|Engraulidae
Anchoa marinii|FARG611-09|Engraulidae
Engraulis anchoita|FARG029-06|Engraulidae
Engraulis anchoita|FARG026-06|Engraulidae
Engraulis anchoita|FARG027-06|Engraulidae
Anchoa marinii|FARG610-09|Engraulidae
Anchoa marinii|FARG612-09|Engraulidae
Brevoortia aurea|FARG626-09|Clupeidae
Brevoortia aurea|FARG273-07|Clupeidae
Brevoortia aurea|FARG623-09|Clupeidae
Brevoortia aurea|FARG271-07|Clupeidae
Brevoortia aurea|FARG593-09|Clupeidae
Brevoortia aurea|FARG595-09|Clupeidae
Brevoortia aurea|FARG625-09|Clupeidae
Brevoortia aurea|FARG292-07|Clupeidae
Brevoortia aurea|FARG274-07|Clupeidae
Brevoortia aurea|FARG272-07|Clupeidae

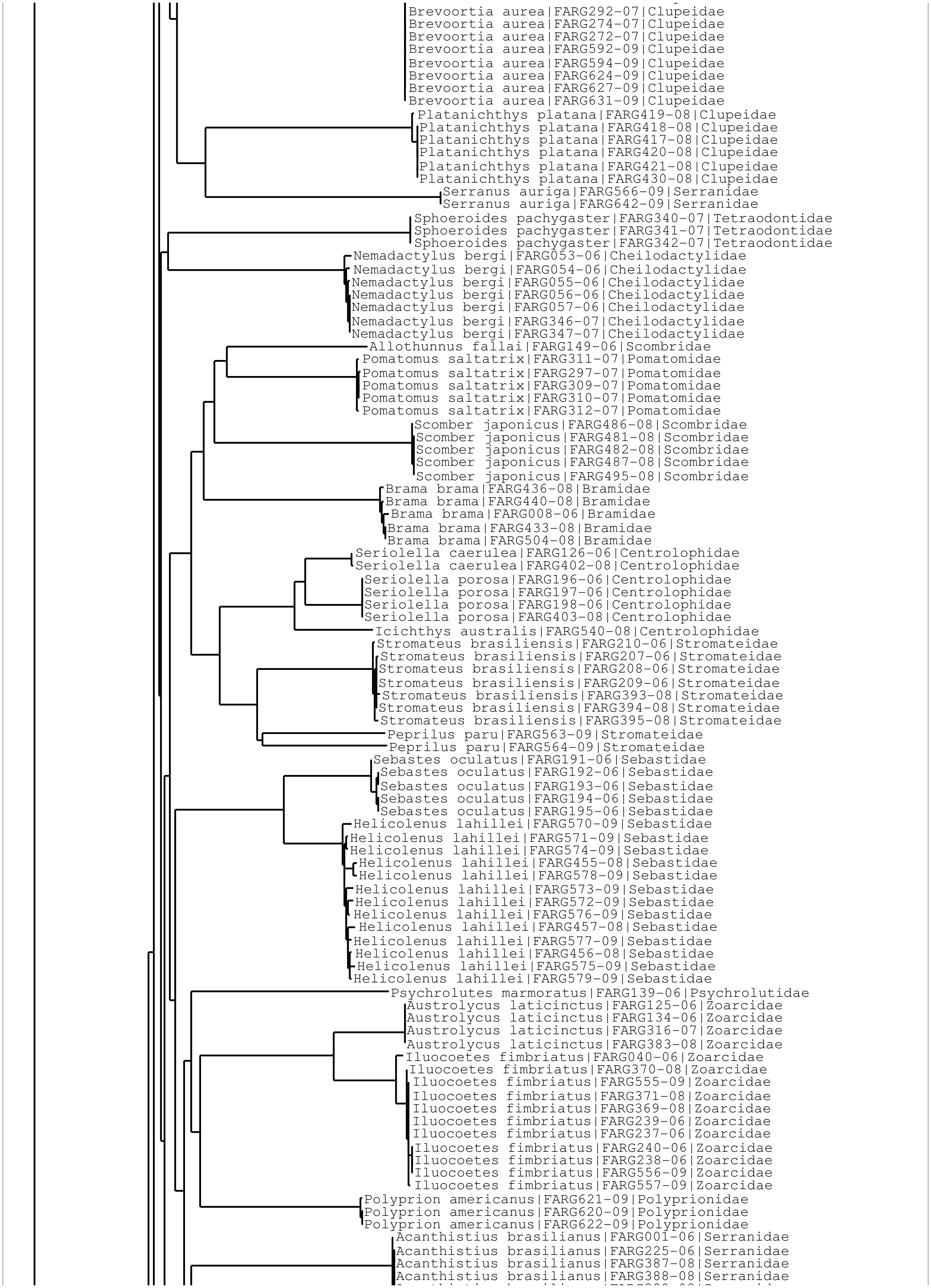
Brevoortia aurea|FARG292-07|Clupeidae
Brevoortia aurea|FARG274-07|Clupeidae
Brevoortia aurea|FARG272-07|Clupeidae
Brevoortia aurea|FARG592-09|Clupeidae
Brevoortia aurea|FARG594-09|Clupeidae
Brevoortia aurea|FARG624-09|Clupeidae
Brevoortia aurea|FARG627-09|Clupeidae
Brevoortia aurea|FARG631-09|Clupeidae
Platanichthys platana|FARG419-08|Clupeidae
Platanichthys platana|FARG418-08|Clupeidae
Platanichthys platana|FARG417-08|Clupeidae
Platanichthys platana|FARG420-08|Clupeidae
Platanichthys platana|FARG421-08|Clupeidae
Platanichthys platana|FARG430-08|Clupeidae
Serranus auriga|FARG566-09|Serranidae
Serranus auriga|FARG642-09|Serranidae
Sphoeroides pachygaster|FARG340-07|Tetraodontidae
Sphoeroides pachygaster|FARG341-07|Tetraodontidae
Sphoeroides pachygaster|FARG342-07|Tetraodontidae
Nemadactylus bergi|FARG053-06|Cheilodactylidae
Nemadactylus bergi|FARG054-06|Cheilodactylidae
Nemadactylus bergi|FARG055-06|Cheilodactylidae
Nemadactylus bergi|FARG056-06|Cheilodactylidae
Nemadactylus bergi|FARG057-06|Cheilodactylidae
Nemadactylus bergi|FARG346-07|Cheilodactylidae
Nemadactylus bergi|FARG347-07|Cheilodactylidae
Allothunnus fallai|FARG149-06|Scombridae
Pomatomus saltatrix|FARG311-07|Pomatomidae
Pomatomus saltatrix|FARG297-07|Pomatomidae
Pomatomus saltatrix|FARG309-07|Pomatomidae
Pomatomus saltatrix|FARG310-07|Pomatomidae
Pomatomus saltatrix|FARG312-07|Pomatomidae
Scomber japonicus|FARG486-08|Scombridae
Scomber japonicus|FARG481-08|Scombridae
Scomber japonicus|FARG482-08|Scombridae
Scomber japonicus|FARG487-08|Scombridae
Scomber japonicus|FARG495-08|Scombridae
Brama brama|FARG436-08|Bramidae
Brama brama|FARG440-08|Bramidae
Brama brama|FARG008-06|Bramidae
Brama brama|FARG433-08|Bramidae
Brama brama|FARG504-08|Bramidae
Seriolella caerulea|FARG126-06|Centrolophidae
Seriolella caerulea|FARG402-08|Centrolophidae
Seriolella porosa|FARG196-06|Centrolophidae
Seriolella porosa|FARG197-06|Centrolophidae
Seriolella porosa|FARG198-06|Centrolophidae
Seriolella porosa|FARG403-08|Centrolophidae
Icichthys australis|FARG540-08|Centrolophidae
Stromateus brasiliensis|FARG210-06|Stromateidae
Stromateus brasiliensis|FARG207-06|Stromateidae
Stromateus brasiliensis|FARG208-06|Stromateidae
Stromateus brasiliensis|FARG209-06|Stromateidae
Stromateus brasiliensis|FARG393-08|Stromateidae
Stromateus brasiliensis|FARG394-08|Stromateidae
Stromateus brasiliensis|FARG395-08|Stromateidae
Peprilus paru|FARG563-09|Stromateidae
Peprilus paru|FARG564-09|Stromateidae
Sebastes oculatus|FARG191-06|Sebastidae
Sebastes oculatus|FARG192-06|Sebastidae
Sebastes oculatus|FARG193-06|Sebastidae
Sebastes oculatus|FARG194-06|Sebastidae
Sebastes oculatus|FARG195-06|Sebastidae
Helicolenus lahillei|FARG570-09|Sebastidae
Helicolenus lahillei|FARG571-09|Sebastidae
Helicolenus lahillei|FARG574-09|Sebastidae
Helicolenus lahillei|FARG455-08|Sebastidae
Helicolenus lahillei|FARG578-09|Sebastidae
Helicolenus lahillei|FARG573-09|Sebastidae
Helicolenus lahillei|FARG572-09|Sebastidae
Helicolenus lahillei|FARG576-09|Sebastidae
Helicolenus lahillei|FARG457-08|Sebastidae
Helicolenus lahillei|FARG577-09|Sebastidae
Helicolenus lahillei|FARG456-08|Sebastidae
Helicolenus lahillei|FARG575-09|Sebastidae
Helicolenus lahillei|FARG579-09|Sebastidae
Psychrolutes marmoratus|FARG139-06|Psychrolutidae
Austrolycus laticinctus|FARG125-06|Zoarcidae
Austrolycus laticinctus|FARG134-06|Zoarcidae
Austrolycus laticinctus|FARG316-07|Zoarcidae
Austrolycus laticinctus|FARG383-08|Zoarcidae
Iluocoetes fimbriatus|FARG040-06|Zoarcidae
Iluocoetes fimbriatus|FARG370-08|Zoarcidae
Iluocoetes fimbriatus|FARG555-09|Zoarcidae
Iluocoetes fimbriatus|FARG371-08|Zoarcidae
Iluocoetes fimbriatus|FARG369-08|Zoarcidae
Iluocoetes fimbriatus|FARG239-06|Zoarcidae
Iluocoetes fimbriatus|FARG237-06|Zoarcidae
Iluocoetes fimbriatus|FARG240-06|Zoarcidae
Iluocoetes fimbriatus|FARG238-06|Zoarcidae
Iluocoetes fimbriatus|FARG556-09|Zoarcidae
Iluocoetes fimbriatus|FARG557-09|Zoarcidae
Polyprion americanus|FARG621-09|Polyprionidae
Polyprion americanus|FARG620-09|Polyprionidae
Polyprion americanus|FARG622-09|Polyprionidae
Acanthistius brasilianus|FARG001-06|Serranidae
Acanthistius brasilianus|FARG225-06|Serranidae
Acanthistius brasilianus|FARG387-08|Serranidae
Acanthistius brasilianus|FARG388-08|Serranidae

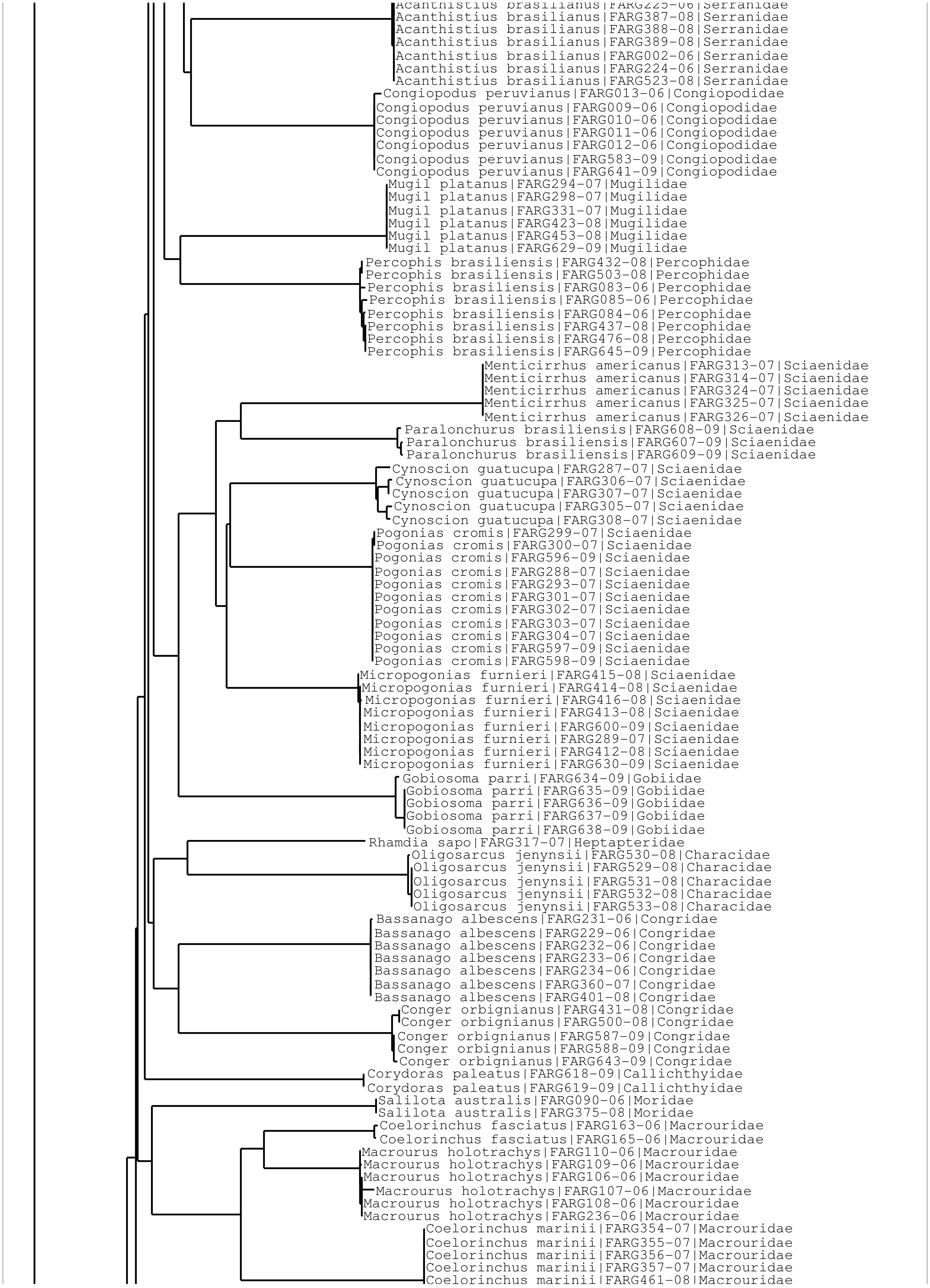
Acanthistius brasilianus|FARG225-06|Serranidae
Acanthistius brasilianus|FARG387-08|Serranidae
Acanthistius brasilianus|FARG388-08|Serranidae
Acanthistius brasilianus|FARG389-08|Serranidae
Acanthistius brasilianus|FARG002-06|Serranidae
Acanthistius brasilianus|FARG224-06|Serranidae
Acanthistius brasilianus|FARG523-08|Serranidae
Congiopodus peruvianus|FARG013-06|Congiopodidae
Congiopodus peruvianus|FARG009-06|Congiopodidae
Congiopodus peruvianus|FARG010-06|Congiopodidae
Congiopodus peruvianus|FARG011-06|Congiopodidae
Congiopodus peruvianus|FARG012-06|Congiopodidae
Congiopodus peruvianus|FARG583-09|Congiopodidae
Congiopodus peruvianus|FARG641-09|Congiopodidae
Mugil platanus|FARG294-07|Mugilidae
Mugil platanus|FARG298-07|Mugilidae
Mugil platanus|FARG331-07|Mugilidae
Mugil platanus|FARG423-08|Mugilidae
Mugil platanus|FARG453-08|Mugilidae
Mugil platanus|FARG629-09|Mugilidae
Percophis brasiliensis|FARG432-08|Percophidae
Percophis brasiliensis|FARG503-08|Percophidae
Percophis brasiliensis|FARG083-06|Percophidae
Percophis brasiliensis|FARG085-06|Percophidae
Percophis brasiliensis|FARG084-06|Percophidae
Percophis brasiliensis|FARG437-08|Percophidae
Percophis brasiliensis|FARG476-08|Percophidae
Percophis brasiliensis|FARG645-09|Percophidae
Menticirrhus americanus|FARG313-07|Sciaenidae
Menticirrhus americanus|FARG314-07|Sciaenidae
Menticirrhus americanus|FARG324-07|Sciaenidae
Menticirrhus americanus|FARG325-07|Sciaenidae
Menticirrhus americanus|FARG326-07|Sciaenidae
Paralonchurus brasiliensis|FARG608-09|Sciaenidae
Paralonchurus brasiliensis|FARG607-09|Sciaenidae
Paralonchurus brasiliensis|FARG609-09|Sciaenidae
Cynoscion guatucupa|FARG287-07|Sciaenidae
Cynoscion guatucupa|FARG306-07|Sciaenidae
Cynoscion guatucupa|FARG307-07|Sciaenidae
Cynoscion guatucupa|FARG305-07|Sciaenidae
Cynoscion guatucupa|FARG308-07|Sciaenidae
Pogonias cromis|FARG299-07|Sciaenidae
Pogonias cromis|FARG300-07|Sciaenidae
Pogonias cromis|FARG596-09|Sciaenidae
Pogonias cromis|FARG288-07|Sciaenidae
Pogonias cromis|FARG293-07|Sciaenidae
Pogonias cromis|FARG301-07|Sciaenidae
Pogonias cromis|FARG302-07|Sciaenidae
Pogonias cromis|FARG303-07|Sciaenidae
Pogonias cromis|FARG304-07|Sciaenidae
Pogonias cromis|FARG597-09|Sciaenidae
Pogonias cromis|FARG598-09|Sciaenidae
Micropogonias furnieri|FARG415-08|Sciaenidae
Micropogonias furnieri|FARG414-08|Sciaenidae
Micropogonias furnieri|FARG416-08|Sciaenidae
Micropogonias furnieri|FARG413-08|Sciaenidae
Micropogonias furnieri|FARG600-09|Sciaenidae
Micropogonias furnieri|FARG289-07|Sciaenidae
Micropogonias furnieri|FARG412-08|Sciaenidae
Micropogonias furnieri|FARG630-09|Sciaenidae
Gobiosoma parri|FARG634-09|Gobiidae
Gobiosoma parri|FARG635-09|Gobiidae
Gobiosoma parri|FARG636-09|Gobiidae
Gobiosoma parri|FARG637-09|Gobiidae
Gobiosoma parri|FARG638-09|Gobiidae
Rhamdia sapo|FARG317-07|Heptapteridae
Oligosarcus jenynsii|FARG530-08|Characidae
Oligosarcus jenynsii|FARG529-08|Characidae
Oligosarcus jenynsii|FARG531-08|Characidae
Oligosarcus jenynsii|FARG532-08|Characidae
Oligosarcus jenynsii|FARG533-08|Characidae
Bassanago albescens|FARG231-06|Congridae
Bassanago albescens|FARG229-06|Congridae
Bassanago albescens|FARG232-06|Congridae
Bassanago albescens|FARG233-06|Congridae
Bassanago albescens|FARG234-06|Congridae
Bassanago albescens|FARG360-07|Congridae
Bassanago albescens|FARG401-08|Congridae
Conger orbignianus|FARG431-08|Congridae
Conger orbignianus|FARG500-08|Congridae
Conger orbignianus|FARG587-09|Congridae
Conger orbignianus|FARG588-09|Congridae
Conger orbignianus|FARG643-09|Congridae
Corydoras paleatus|FARG618-09|Callichthyidae
Corydoras paleatus|FARG619-09|Callichthyidae
Salilota australis|FARG090-06|Moridae
Salilota australis|FARG375-08|Moridae
Coelorinchus fasciatus|FARG163-06|Macrouridae
Coelorinchus fasciatus|FARG165-06|Macrouridae
Macrourus holotrachys|FARG110-06|Macrouridae
Macrourus holotrachys|FARG109-06|Macrouridae
Macrourus holotrachys|FARG106-06|Macrouridae
Macrourus holotrachys|FARG107-06|Macrouridae
Macrourus holotrachys|FARG108-06|Macrouridae
Macrourus holotrachys|FARG236-06|Macrouridae
Coelorinchus marinii|FARG354-07|Macrouridae
Coelorinchus marinii|FARG355-07|Macrouridae
Coelorinchus marinii|FARG356-07|Macrouridae
Coelorinchus marinii|FARG357-07|Macrouridae
Coelorinchus marinii|FARG461-08|Macrouridae

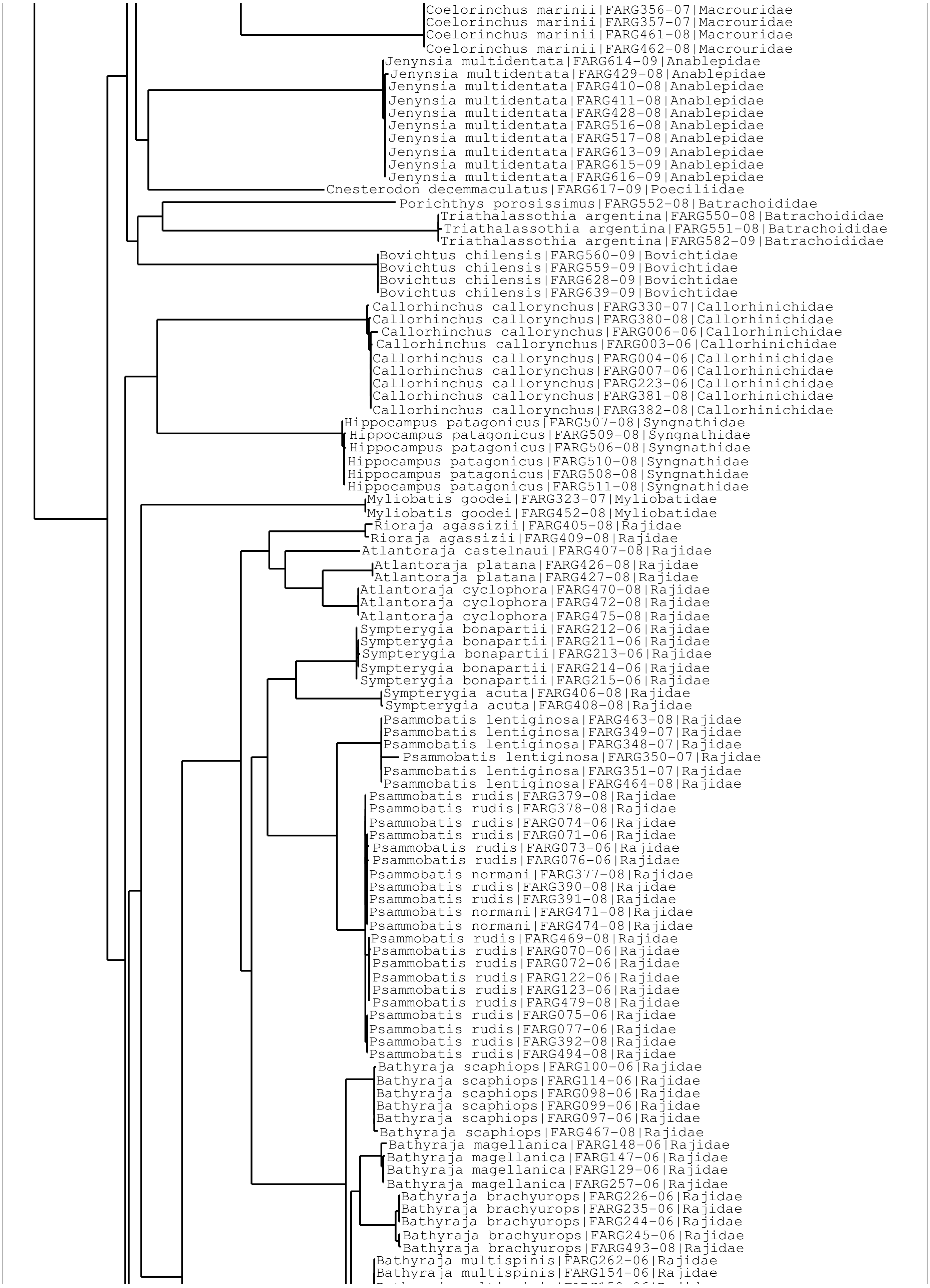
Coelorinchus marinii|FARG356-07|Macrouridae
Coelorinchus marinii|FARG357-07|Macrouridae
Coelorinchus marinii|FARG461-08|Macrouridae
Coelorinchus marinii|FARG462-08|Macrouridae
Jenynsia multidentata|FARG614-09|Anablepidae
Jenynsia multidentata|FARG429-08|Anablepidae
Jenynsia multidentata|FARG410-08|Anablepidae
Jenynsia multidentata|FARG411-08|Anablepidae
Jenynsia multidentata|FARG428-08|Anablepidae
Jenynsia multidentata|FARG516-08|Anablepidae
Jenynsia multidentata|FARG517-08|Anablepidae
Jenynsia multidentata|FARG613-09|Anablepidae
Jenynsia multidentata|FARG615-09|Anablepidae
Jenynsia multidentata|FARG616-09|Anablepidae
Cnesterodon decemmaculatus|FARG617-09|Poeciliidae
Porichthys porosissimus|FARG552-08|Batrachoididae
Triathalassothia argentina|FARG550-08|Batrachoididae
Triathalassothia argentina|FARG551-08|Batrachoididae
Triathalassothia argentina|FARG582-09|Batrachoididae
Bovichtus chilensis|FARG560-09|Bovichtidae
Bovichtus chilensis|FARG559-09|Bovichtidae
Bovichtus chilensis|FARG628-09|Bovichtidae
Bovichtus chilensis|FARG639-09|Bovichtidae
Callorhinchus callorynchus|FARG330-07|Callorhinichidae
Callorhinchus callorynchus|FARG380-08|Callorhinichidae
Callorhinchus callorynchus|FARG006-06|Callorhinichidae
Callorhinchus callorynchus|FARG003-06|Callorhinichidae
Callorhinchus callorynchus|FARG004-06|Callorhinichidae
Callorhinchus callorynchus|FARG007-06|Callorhinichidae
Callorhinchus callorynchus|FARG223-06|Callorhinichidae
Callorhinchus callorynchus|FARG381-08|Callorhinichidae
Callorhinchus callorynchus|FARG382-08|Callorhinichidae
Hippocampus patagonicus|FARG507-08|Syngnathidae
Hippocampus patagonicus|FARG509-08|Syngnathidae
Hippocampus patagonicus|FARG506-08|Syngnathidae
Hippocampus patagonicus|FARG510-08|Syngnathidae
Hippocampus patagonicus|FARG508-08|Syngnathidae
Hippocampus patagonicus|FARG511-08|Syngnathidae
Myliobatis goodei|FARG323-07|Myliobatidae
Myliobatis goodei|FARG452-08|Myliobatidae
Rioraja agassizii|FARG405-08|Rajidae
Rioraja agassizii|FARG409-08|Rajidae
Atlantoraja castelnaui|FARG407-08|Rajidae
Atlantoraja platana|FARG426-08|Rajidae
Atlantoraja platana|FARG427-08|Rajidae
Atlantoraja cyclophora|FARG470-08|Rajidae
Atlantoraja cyclophora|FARG472-08|Rajidae
Atlantoraja cyclophora|FARG475-08|Rajidae
Sympterygia bonapartii|FARG212-06|Rajidae
Sympterygia bonapartii|FARG211-06|Rajidae
Sympterygia bonapartii|FARG213-06|Rajidae
Sympterygia bonapartii|FARG214-06|Rajidae
Sympterygia bonapartii|FARG215-06|Rajidae
Sympterygia acuta|FARG406-08|Rajidae
Sympterygia acuta|FARG408-08|Rajidae
Psammobatis lentiginosa|FARG463-08|Rajidae
Psammobatis lentiginosa|FARG349-07|Rajidae
Psammobatis lentiginosa|FARG348-07|Rajidae
Psammobatis lentiginosa|FARG350-07|Rajidae
Psammobatis lentiginosa|FARG351-07|Rajidae
Psammobatis lentiginosa|FARG464-08|Rajidae
Psammobatis rudis|FARG379-08|Rajidae
Psammobatis rudis|FARG378-08|Rajidae
Psammobatis rudis|FARG074-06|Rajidae
Psammobatis rudis|FARG071-06|Rajidae
Psammobatis rudis|FARG073-06|Rajidae
Psammobatis rudis|FARG076-06|Rajidae
Psammobatis normani|FARG377-08|Rajidae
Psammobatis rudis|FARG390-08|Rajidae
Psammobatis rudis|FARG391-08|Rajidae
Psammobatis normani|FARG471-08|Rajidae
Psammobatis normani|FARG474-08|Rajidae
Psammobatis rudis|FARG469-08|Rajidae
Psammobatis rudis|FARG070-06|Rajidae
Psammobatis rudis|FARG072-06|Rajidae
Psammobatis rudis|FARG122-06|Rajidae
Psammobatis rudis|FARG123-06|Rajidae
Psammobatis rudis|FARG479-08|Rajidae
Psammobatis rudis|FARG075-06|Rajidae
Psammobatis rudis|FARG077-06|Rajidae
Psammobatis rudis|FARG392-08|Rajidae
Psammobatis rudis|FARG494-08|Rajidae
Bathyraja scaphiops|FARG100-06|Rajidae
Bathyraja scaphiops|FARG114-06|Rajidae
Bathyraja scaphiops|FARG098-06|Rajidae
Bathyraja scaphiops|FARG099-06|Rajidae
Bathyraja scaphiops|FARG097-06|Rajidae
Bathyraja scaphiops|FARG467-08|Rajidae
Bathyraja magellanica|FARG148-06|Rajidae
Bathyraja magellanica|FARG147-06|Rajidae
Bathyraja magellanica|FARG129-06|Rajidae
Bathyraja magellanica|FARG257-06|Rajidae
Bathyraja brachyurops|FARG226-06|Rajidae
Bathyraja brachyurops|FARG235-06|Rajidae
Bathyraja brachyurops|FARG244-06|Rajidae
Bathyraja brachyurops|FARG245-06|Rajidae
Bathyraja brachyurops|FARG493-08|Rajidae
Bathyraja multispinis|FARG262-06|Rajidae
Bathyraja multispinis|FARG154-06|Rajidae

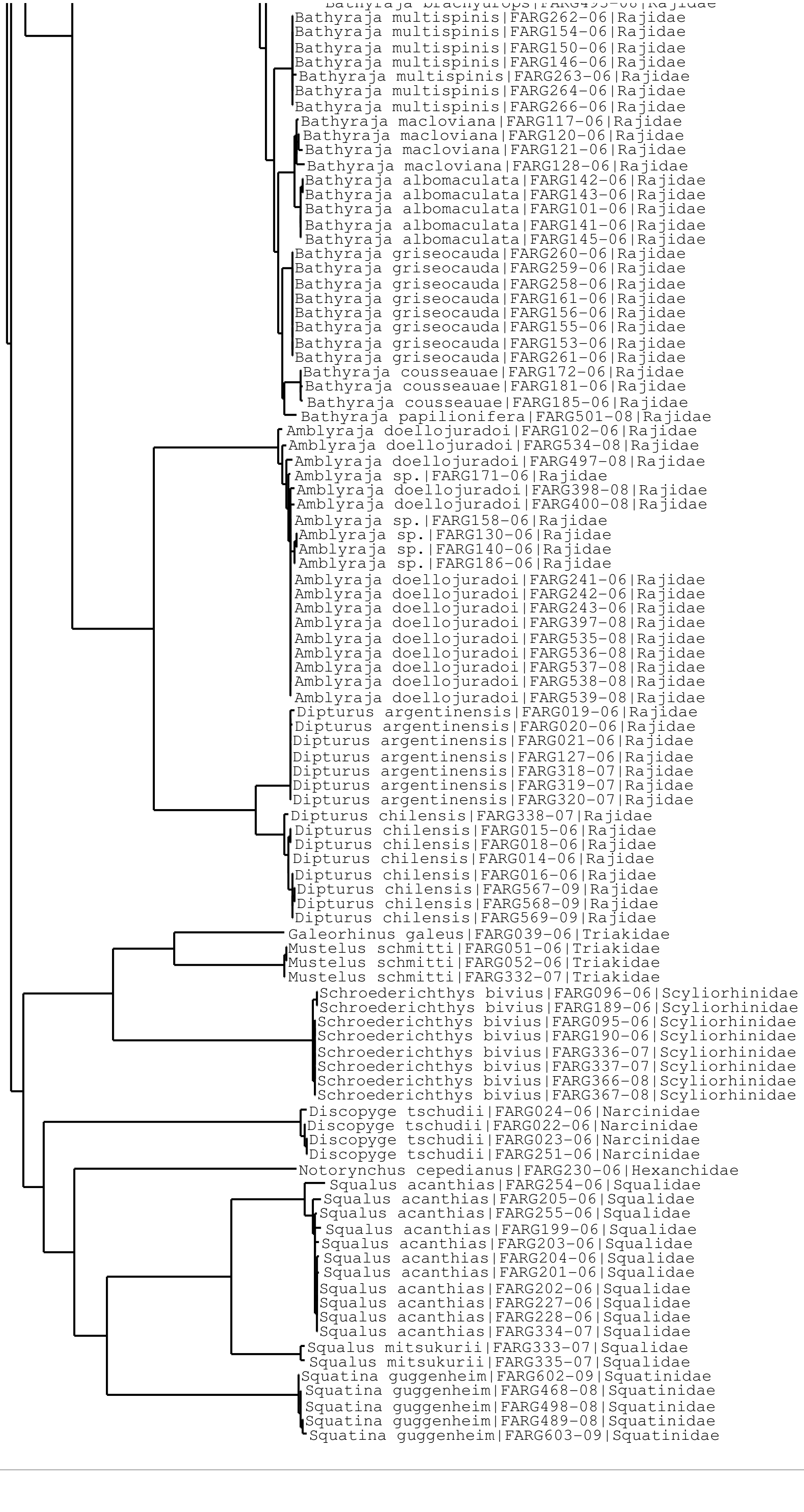
Bathyraja multispinis|FARG262-06|Rajidae
Bathyraja multispinis|FARG154-06|Rajidae
Bathyraja multispinis|FARG150-06|Rajidae
Bathyraja multispinis|FARG146-06|Rajidae
Bathyraja multispinis|FARG263-06|Rajidae
Bathyraja multispinis|FARG264-06|Rajidae
Bathyraja multispinis|FARG266-06|Rajidae
Bathyraja macloviana|FARG117-06|Rajidae
Bathyraja macloviana|FARG120-06|Rajidae
Bathyraja macloviana|FARG121-06|Rajidae
Bathyraja macloviana|FARG128-06|Rajidae
Bathyraja albomaculata|FARG142-06|Rajidae
Bathyraja albomaculata|FARG143-06|Rajidae
Bathyraja albomaculata|FARG101-06|Rajidae
Bathyraja albomaculata|FARG141-06|Rajidae
Bathyraja albomaculata|FARG145-06|Rajidae
Bathyraja griseocauda|FARG260-06|Rajidae
Bathyraja griseocauda|FARG259-06|Rajidae
Bathyraja griseocauda|FARG258-06|Rajidae
Bathyraja griseocauda|FARG161-06|Rajidae
Bathyraja griseocauda|FARG156-06|Rajidae
Bathyraja griseocauda|FARG155-06|Rajidae
Bathyraja griseocauda|FARG153-06|Rajidae
Bathyraja griseocauda|FARG261-06|Rajidae
Bathyraja cousseauae|FARG172-06|Rajidae
Bathyraja cousseauae|FARG181-06|Rajidae
Bathyraja cousseauae|FARG185-06|Rajidae
Bathyraja papilionifera|FARG501-08|Rajidae
Amblyraja doellojuradoi|FARG102-06|Rajidae
Amblyraja doellojuradoi|FARG534-08|Rajidae
Amblyraja doellojuradoi|FARG497-08|Rajidae
Amblyraja sp.|FARG171-06|Rajidae
Amblyraja doellojuradoi|FARG398-08|Rajidae
Amblyraja doellojuradoi|FARG400-08|Rajidae
Amblyraja sp.|FARG158-06|Rajidae
Amblyraja sp.|FARG130-06|Rajidae
Amblyraja sp.|FARG140-06|Rajidae
Amblyraja sp.|FARG186-06|Rajidae
Amblyraja doellojuradoi|FARG241-06|Rajidae
Amblyraja doellojuradoi|FARG242-06|Rajidae
Amblyraja doellojuradoi|FARG243-06|Rajidae
Amblyraja doellojuradoi|FARG397-08|Rajidae
Amblyraja doellojuradoi|FARG535-08|Rajidae
Amblyraja doellojuradoi|FARG536-08|Rajidae
Amblyraja doellojuradoi|FARG537-08|Rajidae
Amblyraja doellojuradoi|FARG538-08|Rajidae
Amblyraja doellojuradoi|FARG539-08|Rajidae
Dipturus argentinensis|FARG019-06|Rajidae
Dipturus argentinensis|FARG020-06|Rajidae
Dipturus argentinensis|FARG021-06|Rajidae
Dipturus argentinensis|FARG127-06|Rajidae
Dipturus argentinensis|FARG318-07|Rajidae
Dipturus argentinensis|FARG319-07|Rajidae
Dipturus argentinensis|FARG320-07|Rajidae
Dipturus chilensis|FARG338-07|Rajidae
Dipturus chilensis|FARG015-06|Rajidae
Dipturus chilensis|FARG018-06|Rajidae
Dipturus chilensis|FARG014-06|Rajidae
Dipturus chilensis|FARG016-06|Rajidae
Dipturus chilensis|FARG567-09|Rajidae
Dipturus chilensis|FARG568-09|Rajidae
Dipturus chilensis|FARG569-09|Rajidae
Galeorhinus galeus|FARG039-06|Triakidae
Mustelus schmitti|FARG051-06|Triakidae
Mustelus schmitti|FARG052-06|Triakidae
Mustelus schmitti|FARG332-07|Triakidae
Schroederichthys bivius|FARG096-06|Scyliorhinidae
Schroederichthys bivius|FARG189-06|Scyliorhinidae
Schroederichthys bivius|FARG095-06|Scyliorhinidae
Schroederichthys bivius|FARG190-06|Scyliorhinidae
Schroederichthys bivius|FARG336-07|Scyliorhinidae
Schroederichthys bivius|FARG337-07|Scyliorhinidae
Schroederichthys bivius|FARG366-08|Scyliorhinidae
Schroederichthys bivius|FARG367-08|Scyliorhinidae
Discopyge tschudii|FARG024-06|Narcinidae
Discopyge tschudii|FARG022-06|Narcinidae
Discopyge tschudii|FARG023-06|Narcinidae
Discopyge tschudii|FARG251-06|Narcinidae
Notorynchus cepedianus|FARG230-06|Hexanchidae
Squalus acanthias|FARG254-06|Squalidae
Squalus acanthias|FARG205-06|Squalidae
Squalus acanthias|FARG255-06|Squalidae
Squalus acanthias|FARG199-06|Squalidae
Squalus acanthias|FARG203-06|Squalidae
Squalus acanthias|FARG204-06|Squalidae
Squalus acanthias|FARG201-06|Squalidae
Squalus acanthias|FARG202-06|Squalidae
Squalus acanthias|FARG227-06|Squalidae
Squalus acanthias|FARG228-06|Squalidae
Squalus acanthias|FARG334-07|Squalidae
Squalus mitsukurii|FARG333-07|Squalidae
Squalus mitsukurii|FARG335-07|Squalidae
Squatina guggenheim|FARG602-09|Squatinidae
Squatina guggenheim|FARG468-08|Squatinidae
Squatina guggenheim|FARG498-08|Squatinidae
Squatina guggenheim|FARG489-08|Squatinidae
Squatina guggenheim|FARG603-09|Squatinidae
